# Augmented reality and virtual reality displays: emerging technologies and future perspectives

**DOI:** 10.1038/s41377-021-00658-8

**Published:** 2021-10-25

**Authors:** Jianghao Xiong, En-Lin Hsiang, Ziqian He, Tao Zhan, Shin-Tson Wu

**Affiliations:** College of Optics and Photonics, University of Central Florida, Orlando, FL 32816 USA

**Keywords:** Displays, Liquid crystals

## Abstract

With rapid advances in high-speed communication and computation, augmented reality (AR) and virtual reality (VR) are emerging as next-generation display platforms for deeper human-digital interactions. Nonetheless, to simultaneously match the exceptional performance of human vision and keep the near-eye display module compact and lightweight imposes unprecedented challenges on optical engineering. Fortunately, recent progress in holographic optical elements (HOEs) and lithography-enabled devices provide innovative ways to tackle these obstacles in AR and VR that are otherwise difficult with traditional optics. In this review, we begin with introducing the basic structures of AR and VR headsets, and then describing the operation principles of various HOEs and lithography-enabled devices. Their properties are analyzed in detail, including strong selectivity on wavelength and incident angle, and multiplexing ability of volume HOEs, polarization dependency and active switching of liquid crystal HOEs, device fabrication, and properties of micro-LEDs (light-emitting diodes), and large design freedoms of metasurfaces. Afterwards, we discuss how these devices help enhance the AR and VR performance, with detailed description and analysis of some state-of-the-art architectures. Finally, we cast a perspective on potential developments and research directions of these photonic devices for future AR and VR displays.

## Abstract (web summary)

Emerging holographic optical elements and lithography-based devices are enhancing the performances of augmented reality and virtual reality displays with glasses-like form factor.

## Graphical abstract

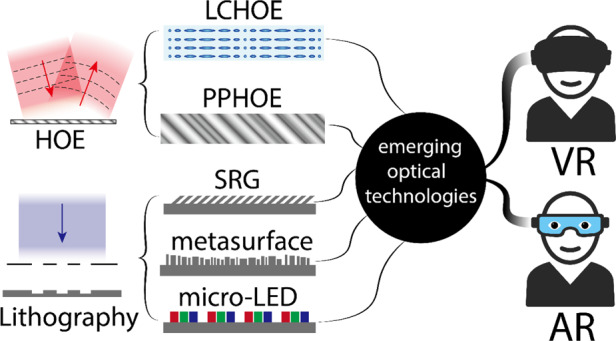

## Introduction

Recent advances in high-speed communication and miniature mobile computing platforms have escalated a strong demand for deeper human-digital interactions beyond traditional flat panel displays. Augmented reality (AR) and virtual reality (VR) headsets^1,2^ are emerging as next-generation interactive displays with the ability to provide vivid three-dimensional (3D) visual experiences. Their useful applications include education, healthcare, engineering, and gaming, just to name a few^3–5^. VR embraces a total immersive experience, while AR promotes the interaction between user, digital contents, and real world, therefore displaying virtual images while remaining see-through capability. In terms of display performance, AR and VR face several common challenges to satisfy demanding human vision requirements, including field of view (FoV), eyebox, angular resolution, dynamic range, and correct depth cue, etc. Another pressing demand, although not directly related to optical performance, is ergonomics. To provide a user-friendly wearing experience, AR and VR should be lightweight and ideally have a compact, glasses-like form factor. The above-mentioned requirements, nonetheless, often entail several tradeoff relations with one another, which makes the design of high-performance AR/VR glasses/headsets particularly challenging.

**Fig. 1:**
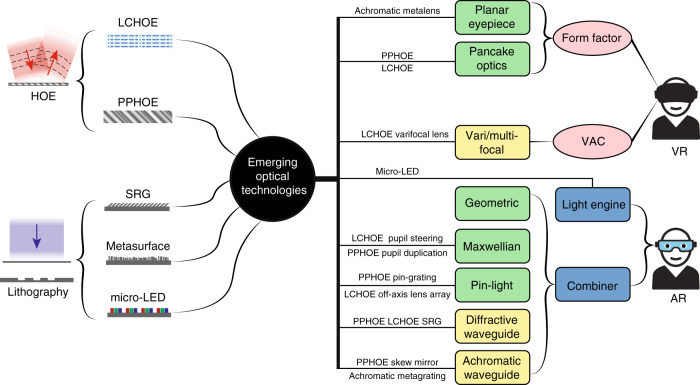
Schematic of some emerging optical technologies applied in AR/VR. The left side illustrates HOEs and lithography-based devices. The right side shows the challenges in VR and architectures in AR, and how the emerging technologies can be applied

In the 1990s, AR/VR experienced the first boom, which quickly subsided due to the lack of eligible hardware and digital content^6^. Over the past decade, the concept of immersive displays was revisited and received a new round of excitement. Emerging technologies like holography and lithography have greatly reshaped the AR/VR display systems. In this article, we firstly review the basic requirements of AR/VR displays and their associated challenges. Then, we briefly describe the properties of two emerging technologies: holographic optical elements (HOEs) and lithography-based devices (Fig. 1). Next, we separately introduce VR and AR systems because of their different device structures and requirements. For the immersive VR system, the major challenges and how these emerging technologies help mitigate the problems will be discussed. For the see-through AR system, we firstly review the present status of light engines and introduce some architectures for the optical combiners. Performance summaries on microdisplay light engines and optical combiners will be provided, that serve as a comprehensive overview of the current AR display systems.

## Key parameters of AR and VR displays

AR and VR displays face several common challenges to satisfy the demanding human vision requirements, such as FoV, eyebox, angular resolution, dynamic range, and correct depth cue, etc. These requirements often exhibit tradeoffs with one another. Before diving into detailed relations, it is beneficial to review the basic definitions of the above-mentioned display parameters.

### Definition of parameters

**Fig. 2:**
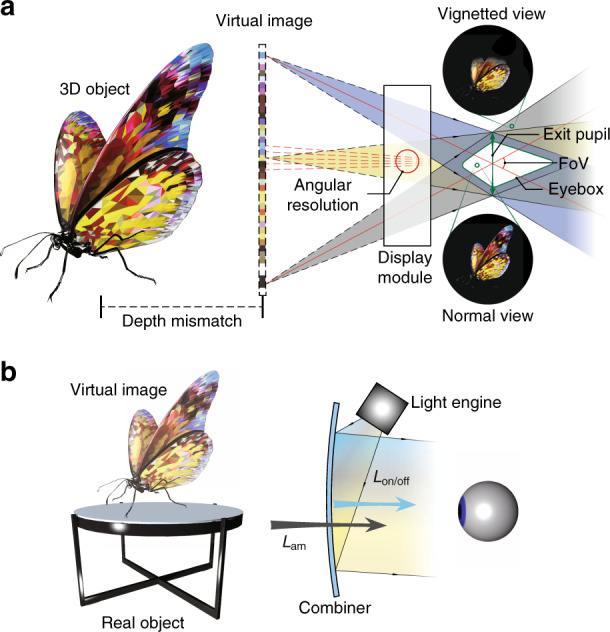
Illustration of display parameters. **a** Schematic of a VR display defining FoV, exit pupil, eyebox, angular resolution, and accommodation cue mismatch. **b** Sketch of an AR display illustrating ACR

Taking a VR system (Fig. 2a) as an example. The light emitting from the display module is projected to a FoV, which can be translated to the size of the image perceived by the viewer. For reference, human vision’s horizontal FoV can be as large as 160° for monocular vision and 120° for overlapped binocular vision^6^. The intersection area of ray bundles forms the exit pupil, which is usually correlated with another parameter called eyebox. The eyebox defines the region within which the whole image FoV can be viewed without vignetting. It therefore generally manifests a 3D geometry^7^, whose volume is strongly dependent on the exit pupil size. A larger eyebox offers more tolerance to accommodate the user’s diversified interpupillary distance (IPD) and wiggling of headset when in use. Angular resolution is defined by dividing the total resolution of the display panel by FoV, which measures the sharpness of a perceived image. For reference, a human visual acuity of 20/20 amounts to 1 arcmin angular resolution, or 60 pixels per degree (PPD), which is considered as a common goal for AR and VR displays. Another important feature of a 3D display is depth cue. Depth cue can be induced by displaying two separate images to the left eye and the right eye, which forms the vergence cue. But the fixed depth of the displayed image often mismatches with the actual depth of the intended 3D image, which leads to incorrect accommodation cues. This mismatch causes the so-called vergence-accommodation conflict (VAC), which will be discussed in detail later. One important observation is that the VAC issue may be more serious in AR than VR, because the image in an AR display is directly superimposed onto the real-world with correct depth cues. The image contrast is dependent on the display panel and stray light. To achieve a high dynamic range, the display panel should exhibit high brightness, low dark level, and more than 10-bits of gray levels. Nowadays, the display brightness of a typical VR headset is about 150–200 cd/m^2^ (or nits).

Figure 2b depicts a generic structure of an AR display. The definition of above parameters remains the same. One major difference is the influence of ambient light on the image contrast. For a see-through AR display, ambient contrast ratio (ACR)^8^ is commonly used to quantify the image contrast:

1 $${\mathrm{ACR}}=\frac{{L_{\mathrm{on}} + L_{\mathrm{am}} \cdot T}}{{L_{\mathrm{off}} + L_{\mathrm{am}} \cdot T}}$$

where *L*_on_ (*L*_off_) represents the on (off)-state luminance (unit: nit), *L*_am_ is the ambient luminance, and *T* is the see-through transmittance. In general, ambient light is measured in illuminance (lux). For the convenience of comparison, we convert illuminance to luminance by dividing a factor of π, assuming the emission profile is Lambertian. In a normal living room, the illuminance is about 100 lux (i.e., *L*_am_ ≈ 30 nits), while in a typical office lighting condition, *L*_am_ ≈ 150 nits. For outdoors, on an overcast day, *L*_am_ ≈ 300 nits, and *L*_am_ ≈ 3000 nits on a sunny day. For AR displays, a minimum ACR should be 3:1 for recognizable images, 5:1 for adequate readability, and ≥10:1 for outstanding readability. To make a simple estimate without considering all the optical losses, to achieve ACR = 10:1 in a sunny day (~3000 nits), the display needs to deliver a brightness of at least 30,000 nits. This imposes big challenges in finding a high brightness microdisplay and designing a low loss optical combiner.

### Tradeoffs and potential solutions

Next, let us briefly review the tradeoff relations mentioned earlier. To begin with, a larger FoV leads to a lower angular resolution for a given display resolution. In theory, to overcome this tradeoff only requires a high-resolution-display source, along with high-quality optics to support the corresponding modulation transfer function (MTF). To attain 60 PPD across 100° FoV requires a 6K resolution for each eye. This may be realizable in VR headsets because a large display panel, say 2–3 inches, can still accommodate a high resolution with acceptable manufacture cost. However, for a glasses-like wearable AR display, the conflict between small display size and the high solution becomes obvious as further shrinking the pixel size of a microdisplay is challenging.

To circumvent this issue, the concept of the foveated display is proposed^9–13^. The idea is based on that the human eye only has high visual acuity in the central fovea region, which accounts for about 10° FoV. If the high-resolution image is only projected to fovea while the peripheral image remains low resolution, then a microdisplay with 2K resolution can satisfy the need. Regarding the implementation method of foveated display, a straightforward way is to optically combine two display sources^9–11^: one for foveal and one for peripheral FoV. This approach can be regarded as spatial multiplexing of displays. Alternatively, time-multiplexing can also be adopted, by temporally changing the optical path to produce different magnification factors for the corresponding FoV^12^. Finally, another approach without multiplexing is to use a specially designed lens with intended distortion to achieve non-uniform resolution density^13^. Aside from the implementation of foveation, another great challenge is to dynamically steer the foveated region as the viewer’s eye moves. This task is strongly related to pupil steering, which will be discussed in detail later.

A larger eyebox or FoV usually decreases the image brightness, which often lowers the ACR. This is exactly the case for a waveguide AR system with exit pupil expansion (EPE) while operating under a strong ambient light. To improve ACR, one approach is to dynamically adjust the transmittance with a tunable dimmer^14,15^. Another solution is to directly boost the image brightness with a high luminance microdisplay and an efficient combiner optics. Details of this topic will be discussed in the light engine section.

Another tradeoff of FoV and eyebox in geometric optical systems results from the conservation of etendue (or optical invariant). To increase the system etendue requires a larger optics, which in turn compromises the form factor. Finally, to address the VAC issue, the display system needs to generate a proper accommodation cue, which often requires the modulation of image depth or wavefront, neither of which can be easily achieved in a traditional geometric optical system. While remarkable progresses have been made to adopt freeform surfaces^16–18^, to further advance AR and VR systems requires additional novel optics with a higher degree of freedom in structure design and light modulation. Moreover, the employed optics should be thin and lightweight. To mitigate the above-mentioned challenges, diffractive optics is a strong contender. Unlike geometric optics relying on curved surfaces to refract or reflect light, diffractive optics only requires a thin layer of several micrometers to establish efficient light diffractions. Two major types of diffractive optics are HOEs based on wavefront recording and manually written devices like surface relief gratings (SRGs) based on lithography. While SRGs have large design freedoms of local grating geometry, a recent publication^19^ indicates the combination of HOE and freeform optics can also offer a great potential for arbitrary wavefront generation. Furthermore, the advances in lithography have also enabled optical metasurfaces beyond diffractive and refractive optics, and miniature display panels like micro-LED (light-emitting diode). These devices hold the potential to boost the performance of current AR/VR displays, while keeping a lightweight and compact form factor.

## Formation and properties of HOEs

HOE generally refers to a recorded hologram that reproduces the original light wavefront. The concept of holography is proposed by Dennis Gabor^20^, which refers to the process of recording a wavefront in a medium (hologram) and later reconstructing it with a reference beam. Early holography uses intensity-sensitive recording materials like silver halide emulsion, dichromated gelatin, and photopolymer^21^. Among them, photopolymer stands out due to its easy fabrication and ability to capture high-fidelity patterns^22,23^. It has therefore found extensive applications like holographic data storage^23^ and display^24,25^. Photopolymer HOEs (PPHOEs) have a relatively small refractive index modulation and therefore exhibits a strong selectivity on the wavelength and incident angle. Another feature of PPHOE is that several holograms can be recorded into a photopolymer film by consecutive exposures. Later, liquid-crystal holographic optical elements (LCHOEs) based on photoalignment polarization holography have also been developed^25,26^. Due to the inherent anisotropic property of liquid crystals, LCHOEs are extremely sensitive to the polarization state of the input light. This feature, combined with the polarization modulation ability of liquid crystal devices, offers a new possibility for dynamic wavefront modulation in display systems.

## PPHOE

**Fig. 3:**
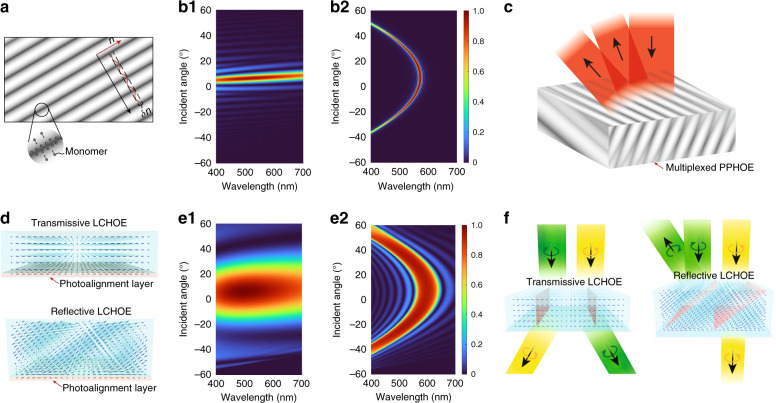
Formation and properties of HOEs. **a** Schematic of the formation of PPHOE. Simulated efficiency plots for **b1** transmissive and **b2** reflective PPHOEs. **c** Working principle of multiplexed PPHOE. **d** Formation and molecular configurations of LCHOEs. Simulated efficiency plots for **e1** transmissive and **e2** reflective LCHOEs. **f** Illustration of polarization dependency of LCHOEs

The formation of PPHOE is illustrated in Fig. 3a. When exposed to an interfering field with high-and-low intensity fringes, monomers tend to move toward bright fringes due to the higher local monomer-consumption rate. As a result, the density and refractive index is slightly larger in bright regions. Note the index modulation δ*n* here is defined as the difference between the maximum and minimum refractive indices, which may be twice the value in other definitions^27^. The index modulation δ*n* is typically in the range of 0–0.06. To understand the optical properties of PPHOE, we simulate a transmissive grating and a reflective grating using rigorous coupled-wave analysis (RCWA)^28,29^ and plot the results in Fig. 3b. Details of grating configuration can be found in Table S1. Here, the reason for only simulating gratings is that for a general HOE, the local region can be treated as a grating. The observation of gratings can therefore offer a general insight of HOEs. For a transmissive grating, its angular bandwidth (efficiency > 80%) is around 5° (*λ* = 550 nm), while the spectral band is relatively broad, with bandwidth around 175 nm (7° incidence). For a reflective grating, its spectral band is narrow, with bandwidth around 10 nm. The angular bandwidth varies with the wavelength, ranging from 2° to 20°. The strong selectivity of PPHOE on wavelength and incident angle is directly related to its small δ*n*, which can be adjusted by controlling the exposure dosage.

A distinctive feature of PPHOE is the ability to multiplex several holograms into one film sample. If the exposure dosage of a recording process is controlled so that the monomers are not completely depleted in the first exposure, the remaining monomers can continue to form another hologram in the following recording process. Because the total amount of monomer is fixed, there is usually an efficiency tradeoff between multiplexed holograms. The final film sample would exhibit the wavefront modulation functions of multiple holograms (Fig. 3c).

## LCHOE

Liquid crystals have also been used to form HOEs. LCHOEs can generally be categorized into volume-recording type and surface-alignment type. Volume-recording type LCHOEs are either based on early polarization holography recordings with azo-polymer^30,31^, or holographic polymer-dispersed liquid crystals (HPDLCs)^32,33^ formed by liquid-crystal-doped photopolymer. Surface-alignment type LCHOEs are based on photoalignment polarization holography (PAPH)^34^. The first step is to record the desired polarization pattern in a thin photoalignment layer, and the second step is to use it to align the bulk liquid crystal^25,35^. Due to the simple fabrication process, high efficiency, and low scattering from liquid crystal’s self-assembly nature, surface-alignment type LCHOEs based on PAPH have recently attracted increasing interest in applications like near-eye displays. Here, we shall focus on this type of surface-alignment LCHOE and refer to it as LCHOE thereafter for simplicity.

The formation of LCHOEs is illustrated in Fig. 3d. The information of the wavefront and the local diffraction pattern is recorded in a thin photoalignment layer. The volume liquid crystal deposited on the photoalignment layer, depending on whether it is nematic liquid crystal or cholesteric liquid crystal (CLC), forms a transmissive or a reflective LCHOE. In a transmissive LCHOE, the bulk nematic liquid crystal molecules generally follow the pattern of the bottom alignment layer. The smallest allowable pattern period is governed by the liquid crystal distortion-free energy model, which predicts the pattern period should generally be larger than sample thickness^36,37^. This results in a maximum diffraction angle under 20°. On the other hand, in a reflective LCHOE^38,39^, the bulk CLC molecules form a stable helical structure, which is tilted to match the *k*-vector of the bottom pattern. The structure exhibits a very low distorted free energy^40,41^ and can accommodate a pattern period that is small enough to diffract light into the total internal reflection (TIR) of a glass substrate.

The diffraction property of LCHOEs is shown in Fig. 3e. The maximum refractive index modulation of LCHOE is equal to the liquid crystal birefringence (Δ*n*), which may vary from 0.04 to 0.5, depending on the molecular conjugation^42,43^. The birefringence used in our simulation is Δ*n* = 0.15. Compared to PPHOEs, the angular and spectral bandwidths are significantly larger for both transmissive and reflective LCHOEs. For a transmissive LCHOE, its angular bandwidth is around 20° (*λ* = 550 nm), while the spectral bandwidth is around 300 nm (7° incidence). For a reflective LCHOE, its spectral bandwidth is around 80 nm and angular bandwidth could vary from 15° to 50°, depending on the wavelength.

The anisotropic nature of liquid crystal leads to LCHOE’s unique polarization-dependent response to an incident light. As depicted in Fig. 3f, for a transmissive LCHOE the accumulated phase is opposite for the conjugated left-handed circular polarization (LCP) and right-handed circular polarization (RCP) states, leading to reversed diffraction directions. For a reflective LCHOE, the polarization dependency is similar to that of a normal CLC. For the circular polarization with the same handedness as the helical structure of CLC, the diffraction is strong. For the opposite circular polarization, the diffraction is negligible.

Another distinctive property of liquid crystal is its dynamic response to an external voltage. The LC reorientation can be controlled with a relatively low voltage (<10 V_rms_) and the response time is on the order of milliseconds, depending mainly on the LC viscosity and layer thickness. Methods to dynamically control LCHOEs can be categorized as active addressing and passive addressing, which can be achieved by either directly switching the LCHOE or modulating the polarization state with an active waveplate. Detailed addressing methods will be described in the VAC section.

### Lithography-enabled devices

Lithography technologies are used to create arbitrary patterns on wafers, which lays the foundation of the modern integrated circuit industry^44^. Photolithography is suitable for mass production while electron/ion beam lithography is usually used to create photomask for photolithography or to write structures with nanometer-scale feature size. Recent advances in lithography have enabled engineered structures like optical metasurfaces^45^, SRGs^46^, as well as micro-LED displays^47^. Metasurfaces exhibit a remarkable design freedom by varying the shape of meta-atoms, which can be utilized to achieve novel functions like achromatic focus^48^ and beam steering^49^. Similarly, SRGs also offer a large design freedom by manipulating the geometry of local grating regions to realize desired optical properties. On the other hand, micro-LED exhibits several unique features, such as ultrahigh peak brightness, small aperture ratio, excellent stability, and nanosecond response time, etc. As a result, micro-LED is a promising candidate for AR and VR systems for achieving high ACR and high frame rate for suppressing motion image blurs. In the following section, we will briefly review the fabrication and properties of micro-LEDs and optical modulators like metasurfaces and SRGs.

### Fabrication and properties of micro-LEDs

**Fig. 4:**
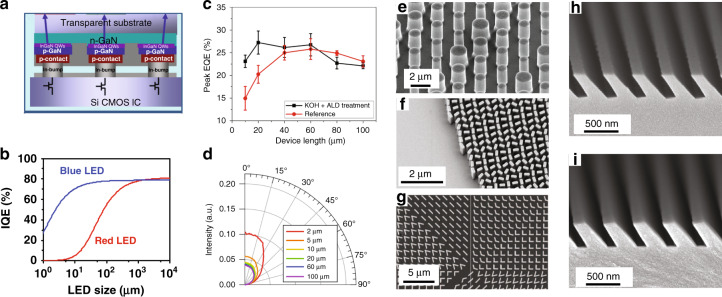
Properties of lithography-enabled micro-LEDs and metasurfaces. **a** Illustration of flip-chip bonding technology. **b** Simulated IQE-LED size relations for red and blue LEDs based on ABC model. **c** Comparison of EQE of different LED sizes with and without KOH and ALD side wall treatment. **d** Angular emission profiles of LEDs with different sizes. Metasurfaces based on **e** resonance-tuning, **f** non-resonance tuning and **g** combination of both. **h** Replication master and **i** replicated SRG based on nanoimprint lithography. Reproduced from **a** ref. ^55^ with permission from AIP Publishing, **b** ref. ^61^ with permission from PNAS, **c** ref. ^66^ with permission from IOP Publishing, **d** ref. ^67^ with permission from AIP Publishing, **e** ref. ^69^ with permission from OSA Publishing **f** ref. ^48^ with permission from AAAS **g** ref. ^70^ with permission from AAAS and **h**, **i** ref. ^85^ with permission from OSA Publishing

LEDs with a chip size larger than 300 μm have been widely used in solid-state lighting and public information displays. Recently, micro-LEDs with chip sizes <5 μm have been demonstrated^50^. The first micro-LED disc with a diameter of about 12 µm was demonstrated in 2000^51^. After that, a single color (blue or green) LED microdisplay was demonstrated in 2012^52^. The high peak brightness, fast response time, true dark state, and long lifetime of micro-LEDs are attractive for display applications. Therefore, many companies have since released their micro-LED prototypes or products, ranging from large-size TVs to small-size microdisplays for AR/VR applications^53,54^. Here, we focus on micro-LEDs for near-eye display applications. Regarding the fabrication of micro-LEDs, through the metal-organic chemical vapor deposition (MOCVD) method, the AlGaInP epitaxial layer is grown on GaAs substrate for red LEDs, and GaN epitaxial layers on sapphire substrate for green and blue LEDs. Next, a photolithography process is applied to define the mesa and deposit electrodes. To drive the LED array, the fabricated micro-LEDs are transferred to a CMOS (complementary metal oxide semiconductor) driver board. For a small size (<2 inches) microdisplay used in AR or VR, the precision of the pick-and-place transfer process is hard to meet the high-resolution-density (>1000 pixel per inch) requirement. Thus, the main approach to assemble LED chips with driving circuits is flip-chip bonding^50,55–57^, as Fig. 4a depicts. In flip-chip bonding, the mesa and electrode pads should be defined and deposited before the transfer process, while metal bonding balls should be preprocessed on the CMOS substrate. After that, thermal-compression method is used to bond the two wafers together. However, due to the thermal mismatch of LED chip and driving board, as the pixel size decreases, the misalignment between the LED chip and the metal bonding ball on the CMOS substrate becomes serious. In addition, the common n-GaN layer may cause optical crosstalk between pixels, which degrades the image quality. To overcome these issues, the LED epitaxial layer can be firstly metal-bonded with the silicon driver board, followed by the photolithography process to define the LED mesas and electrodes. Without the need for an alignment process, the pixel size can be reduced to <5 µm^50^.

In addition to manufacturing process, the electrical and optical characteristics of LED also depend on the chip size. Generally, due to Shockley-Read-Hall (SRH) non-radiative recombination on the sidewall of active area, a smaller LED chip size results in a lower internal quantum efficiency (IQE), so that the peak IQE driving point will move toward a higher current density due to increased ratio of sidewall surface to active volume^58–60^. In addition, compared to the GaN-based green and blue LEDs, the AlGaInP-based red LEDs with a larger surface recombination and carrier diffusion length suffer a more severe efficiency drop^61,62^. Figure 4b shows the simulated result of IQE drop in relation with the LED chip size of blue and red LEDs based on ABC model^63^. To alleviate the efficiency drop caused by sidewall defects, depositing passivation materials by atomic layer deposition (ALD) or plasma enhanced chemical vapor deposition (PECVD) is proven to be helpful for both GaN and AlGaInP based LEDs^64,65^. In addition, applying KOH (Potassium hydroxide) treatment after ALD can further reduce the EQE drop of micro-LEDs^66^ (Fig. 4c). Small-size LEDs also exhibit some advantages, such as higher light extraction efficiency (LEE). Compared to an 100-µm LED, the LEE of a 2-µm LED increases from 12.2 to 25.1%^67^. Moreover, the radiation pattern of micro-LED is more directional than that of a large-size LED (Fig. 4d). This helps to improve the lens collection efficiency in AR/VR display systems.

### Metasurfaces and SGs

Thanks to the advances in lithography technology, low-loss dielectric metasurfaces working in the visible band have recently emerged as a platform for wavefront shaping^45,48,68^. They consist of an array of subwavelength-spaced structures with individually engineered wavelength-dependent polarization/phase/ amplitude response. In general, the light modulation mechanisms can be classified into resonant tuning^69^ (Fig. 4e), non-resonant tuning^48^ (Fig. 4f), and combination of both^70^ (Fig. 4g). In comparison with non-resonant tuning (based on geometric phase and/or dynamic propagation phase), the resonant tuning (such as Fabry–Pérot resonance, Mie resonance, etc.) is usually associated with a narrower operating bandwidth and a smaller out-of-plane aspect ratio (height/width) of nanostructures. As a result, they are easier to fabricate but more sensitive to fabrication tolerances. For both types, materials with a higher refractive index and lower absorption loss are beneficial to reduce the aspect ratio of nanostructure and improve the device efficiency. To this end, titanium dioxide (TiO_2_) and gallium nitride (GaN) are the major choices for operating in the entire visible band^68,71^. While small-sized metasurfaces (diameter <1 mm) are usually fabricated via electron-beam lithography or focused ion beam milling in the labs, the ability of mass production is the key to their practical adoption. The deep ultraviolet (UV) photolithography has proven its feasibility for reproducing centimeter-size metalenses with decent imaging performance, while it requires multiple steps of etching^72^. Interestingly, the recently developed UV nanoimprint lithography based on a high-index nanocomposite only takes a single step and can obtain an aspect ratio larger than 10, which shows great promise for high-volume production^73^.

The arbitrary wavefront shaping capability and the thinness of the metasurfaces have aroused strong research interests in the development of novel AR/VR prototypes with improved performance. Lee et al. employed nanoimprint lithography to fabricate a centimeter-size, geometric-phase metalens eyepiece for full-color AR displays^74^. Through tailoring its polarization conversion efficiency and stacking with a circular polarizer, the virtual image can be superimposed with the surrounding scene. The large numerical aperture (NA~0.5) of the metalens eyepiece enables a wide FoV (>76°) that conventional optics are difficult to obtain. However, the geometric phase metalens is intrinsically a diffractive lens that also suffers from strong chromatic aberrations. To overcome this issue, an achromatic lens can be designed via simultaneously engineering the group delay and the group delay dispersion^75,76^, which will be described in detail later. Other novel and/or improved near-eye display architectures include metasurface-based contact lens-type AR^77^, achromatic metalens array enabled integral-imaging light field displays^78^, wide FoV lightguide AR with polarization-dependent metagratings^79^, and off-axis projection-type AR with an aberration-corrected metasurface combiner^80–82^. Nevertheless, from the existing AR/VR prototypes, metasurfaces still face a strong tradeoff between numerical aperture (for metalenses), chromatic aberration, monochromatic aberration, efficiency, aperture size, and fabrication complexity.

On the other hand, SRGs are diffractive gratings that have been researched for decades as input/output couplers of waveguides^83,84^. Their surface is composed of corrugated microstructures, and different shapes including binary, blazed, slanted, and even analogue can be designed. The parameters of the corrugated microstructures are determined by the target diffraction order, operation spectral bandwidth, and angular bandwidth. Compared to metasurfaces, SRGs have a much larger feature size and thus can be fabricated via UV photolithography and subsequent etching. They are usually replicated by nanoimprint lithography with appropriate heating and surface treatment. According to a report published a decade ago, SRGs with a height of 300 nm and a slant angle of up to 50° can be faithfully replicated with high yield and reproducibility^85^ (Fig. 4g, h).

### Challenges and solutions of VR displays

The fully immersive nature of VR headset leads to a relatively fixed configuration where the display panel is placed in front of the viewer’s eye and an imaging optics is placed in-between. Regarding the system performance, although inadequate angular resolution still exists in some current VR headsets, the improvement of display panel resolution with advanced fabrication process is expected to solve this issue progressively. Therefore, in the following discussion, we will mainly focus on two major challenges: form factor and 3D cue generation.

### Form factor

**Fig. 5:**
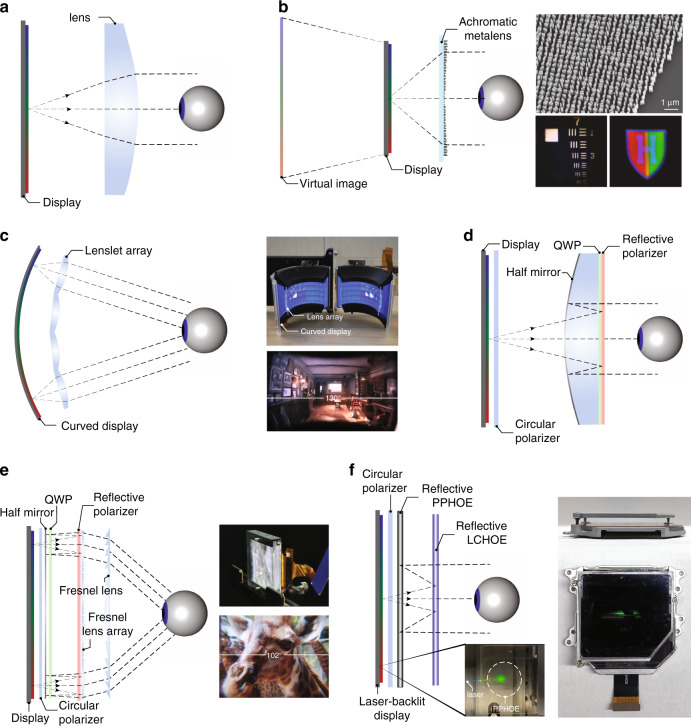
Schemes to reduce the form factor of a VR display. **a** Schematic of a basic VR optical configuration. **b** Achromatic metalens used as VR eyepiece. **c** VR based on curved display and lenslet array. **d** Basic working principle of a VR display based on pancake optics. **e** VR with pancake optics and Fresnel lens array. **f** VR with pancake optics based on purely HOEs. Reprinted from **b** ref. ^87^ under the Creative Commons Attribution 4.0 License. Adapted from **c** ref. ^88^ with permission from IEEE, **e** ref. ^91^ and **f** ref. ^92^ under the Creative Commons Attribution 4.0 License

Compact and lightweight near-eye displays are essential for a comfortable user experience and therefore highly desirable in VR headsets. Current mainstream VR headsets usually have a considerably larger volume than eyeglasses, and most of the volume is just empty. This is because a certain distance is required between the display panel and the viewing optics, which is usually close to the focal length of the lens system as illustrated in Fig. 5a. Conventional VR headsets employ a transmissive lens with ~4 cm focal length to offer a large FoV and eyebox. Fresnel lenses are thinner than conventional ones, but the distance required between the lens and the panel does not change significantly. In addition, the diffraction artifacts and stray light caused by the Fresnel grooves can degrade the image quality, or MTF. Although the resolution density, quantified as pixel per inch (PPI), of current VR headsets is still limited, eventually Fresnel lens will not be an ideal solution when a high PPI display is available. The strong chromatic aberration of Fresnel singlet should also be compensated if a high-quality imaging system is preferred.

It is tempting to replace the refractive elements with a single thin diffractive lens like a transmissive LCHOE. However, the diffractive nature of such a lens will result in serious color aberrations. Interestingly, metalenses can fulfil this objective without color issues. To understand how metalenses achieve achromatic focus, let us first take a glance at the general lens phase profile $\Phi (\omega ,r)$ expanded as a Taylor series^75^:

2 $$\begin{array}{ll}\Phi (\omega ,r) = \varphi _0(\omega )+\frac{{\omega}}{c}\left( {F\left( {\omega} \right) - \sqrt {F\left( {\omega} \right)^2 + r^2} } \right) \\\qquad\quad\, = \Phi (\omega _0 ,r) + \frac{{\partial \Phi (\omega ,r)}}{{\partial \omega }}(\omega - \omega _0) + \frac{{\partial ^2\Phi (\omega ,r)}}{{2\partial \omega ^2}}(\omega - \omega _0)^2 + \cdots \end{array}$$

where $\varphi _0(\omega )$ is the phase at the lens center, $F\left( \omega \right)$ is the focal length as a function of frequency *ω*, *r* is the radial coordinate, and $\omega _0$ is the central operation frequency. To realize achromatic focus, $\partial F{{{\mathrm{/}}}}\partial \omega$ should be zero. With a designed focal length, the group delay $\partial \Phi (\omega ,r){{{\mathrm{/}}}}\partial \omega$ and the group delay dispersion $\partial ^2\Phi (\omega ,r){{{\mathrm{/}}}}\partial \omega ^2$ can be determined, and $\varphi _0(\omega )$ is an auxiliary degree of freedom of the phase profile design. In the design of an achromatic metalens, the group delay is a function of the radial coordinate and monotonically increases with the metalens radius. Many designs have proven that the group delay has a limited variation range^75,76,78,86^. According to Shrestha et al.^86^, there is an inevitable tradeoff between the maximum radius of the metalens, NA, and operation bandwidth. Thus, the reported achromatic metalenses at visible usually have limited lens aperture (e.g., diameter < 250 μm) and NA (e.g., <0.2). Such a tradeoff is undesirable in VR displays, as the eyepiece favors a large clear aperture (inch size) and a reasonably high NA (>0.3) to maintain a wide FoV and a reasonable eye relief^74^.

To overcome this limitation, Li et al.^87^ proposed a novel zone lens method. Unlike the traditional phase Fresnel lens where the zones are determined by the phase reset, the new approach divides the zones by the group delay reset. In this way, the lens aperture and NA can be much enlarged, and the group delay limit is bypassed. A notable side effect of this design is the phase discontinuity at zone boundaries that will contribute to higher-order focusing. Therefore, significant efforts have been conducted to find the optimal zone transition locations and to minimize the phase discontinuities. Using this method, they have demonstrated an impressive 2-mm-diameter metalens with NA = 0.7 and nearly diffraction-limited focusing for the designed wavelengths (488, 532, 658 nm) (Fig. 5b). Such a metalens consists of 681 zones and works for the visible band ranging from 470 to 670 nm, though the focusing efficiency is in the order of 10%. This is a great starting point for the achromatic metalens to be employed as a compact, chromatic-aberration-free eyepiece in near-eye displays. Future challenges are how to further increase the aperture size, correct the off-axis aberrations, and improve the optical efficiency.

Besides replacing the refractive lens with an achromatic metalens, another way to reduce system focal length without decreasing NA is to use a lenslet array^88^. As depicted in Fig. 5c, both the lenslet array and display panel adopt a curved structure. With the latest flexible OLED panel, the display can be easily curved in one dimension. The system exhibits a large diagonal FoV of 180° with an eyebox of 19 by 12 mm. The geometry of each lenslet is optimized separately to achieve an overall performance with high image quality and reduced distortions.

Aside from trying to shorten the system focal length, another way to reduce total track is to fold optical path. Recently, polarization-based folded lenses, also known as pancake optics, are under active development for VR applications^89,90^. Figure 5d depicts the structure of an exemplary singlet pancake VR lens system. The pancake lenses can offer better imaging performance with a compact form factor since there are more degrees of freedom in the design and the actual light path is folded thrice. By using a reflective surface with a positive power, the field curvature of positive refractive lenses can be compensated. Also, the reflective surface has no chromatic aberrations and it contributes considerable optical power to the system. Therefore, the optical power of refractive lenses can be smaller, resulting in an even weaker chromatic aberration. Compared to Fresnel lenses, the pancake lenses have smooth surfaces and much fewer diffraction artifacts and stray light. However, such a pancake lens design is not perfect either, whose major shortcoming is low light efficiency. With two incidences of light on the half mirror, the maximum system efficiency is limited to 25% for a polarized input and 12.5% for an unpolarized input light. Moreover, due to the existence of multiple surfaces in the system, stray light caused by surface reflections and polarization leakage may lead to apparent ghost images. As a result, the catadioptric pancake VR headset usually manifests a darker imagery and lower contrast than the corresponding dioptric VR.

Interestingly, the lenslet and pancake optics can be combined to further reduce the system form. Bang et al.^91^ demonstrated a compact VR system with a pancake optics and a Fresnel lenslet array. The pancake optics serves to fold the optical path between the display panel and the lenslet array (Fig. 5e). Another Fresnel lens is used to collect the light from the lenslet array. The system has a decent horizontal FoV of 102° and an eyebox of 8 mm. However, a certain degree of image discontinuity and crosstalk are still present, which can be improved with further optimizations on the Fresnel lens and the lenslet array.

One step further, replacing all conventional optics in catadioptric VR headset with holographic optics can make the whole system even thinner. Maimone and Wang demonstrated such a lightweight, high-resolution, and ultra-compact VR optical system using purely HOEs^92^. This holographic VR optics was made possible by combining several innovative optical components, including a reflective PPHOE, a reflective LCHOE, and a PPHOE-based directional backlight with laser illumination, as shown in Fig. 5f. Since all the optical power is provided by the HOEs with negligible weight and volume, the total physical thickness can be reduced to <10 mm. Also, unlike conventional bulk optics, the optical power of a HOE is independent of its thickness, only subject to the recording process. Another advantage of using holographic optical devices is that they can be engineered to offer distinct phase profiles for different wavelengths and angles of incidence, adding extra degrees of freedom in optical designs for better imaging performance. Although only a single-color backlight has been demonstrated, such a PPHOE has the potential to achieve full-color laser backlight with multiplexing ability. The PPHOE and LCHOE in the pancake optics can also be optimized at different wavelengths for achieving high-quality full-color images.

### Vergence-accommodation conflict

Conventional VR displays suffer from VAC, which is a common issue for stereoscopic 3D displays^93^. In current VR display modules, the distance between the display panel and the viewing optics is fixed, which means the VR imagery is displayed at a single depth. However, the image contents are generated by parallax rendering in three dimensions, offering distinct images for two eyes. This approach offers a proper stimulus to vergence but completely ignores the accommodation cue, which leads to the well-known VAC that can cause an uncomfortable user experience. Since the beginning of this century, numerous methods have been proposed to solve this critical issue. Methods to produce accommodation cue include multifocal/varifocal display^94^, holographic display^95^, and integral imaging display^96^. Alternatively, elimination of accommodation cue using a Maxwellian-view display^93^ also helps to mitigate the VAC. However, holographic displays and Maxwellian-view displays generally require a totally different optical architecture than current VR systems. They are therefore more suitable for AR displays, which will be discussed later. Integral imaging, on the other hand, has an inherent tradeoff between view number and resolution. For current VR headsets pursuing high resolution to match human visual acuity, it may not be an appealing solution. Therefore, multifocal/varifocal displays that rely on depth modulation is a relatively practical and effective solution for VR headsets. Regarding the working mechanism, multifocal displays present multiple images with different depths to imitate the original 3D scene. Varifocal displays, in contrast, only show one image at each time frame. The image depth matches the viewer’s vergence depth. Nonetheless, the pre-knowledge of the viewer’s vergence depth requires an additional eye-tracking module. Despite different operation principles, a varifocal display can often be converted to a multifocal display as long as the varifocal module has enough modulation bandwidth to support multiple depths in a time frame.

To achieve depth modulation in a VR system, traditional liquid lens^97,98^ with tunable focus suffers from the small aperture and large aberrations. Alvarez lens^99^ is another tunable-focus solution but it requires mechanical adjustment, which adds to system volume and complexity. In comparison, transmissive LCHOEs with polarization dependency can achieve focus adjustment with electronic driving. Its ultra-thinness also satisfies the requirement of small form factors in VR headsets. The diffractive behavior of transmissive LCHOEs is often interpreted by the mechanism of Pancharatnam-Berry phase (also known as geometric phase)^100^. They are therefore often called Pancharatnam-Berry optical elements (PBOEs). The corresponding lens component is referred as Pancharatnam-Berry lens (PBL).

**Fig. 6:**
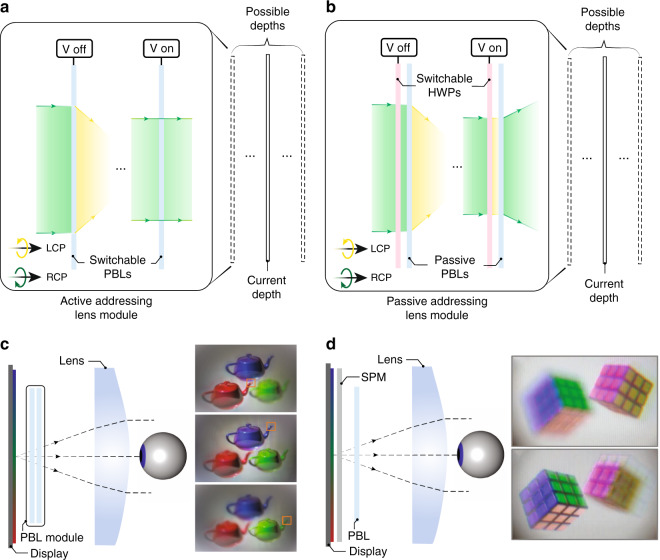
Addressing VAC in VR. Working principles of a depth switching PBL module based on **a** active addressing and **b** passive addressing. **c** A four-depth multifocal display based on time multiplexing. **d** A two-depth multifocal display based on polarization multiplexing. Reproduced from **c** ref. ^103^ with permission from OSA Publishing and **d** ref. ^104^ with permission from OSA Publishing

Two main approaches are used to switch the focus of a PBL, active addressing and passive addressing. In active addressing, the PBL itself (made of LC) can be switched by an applied voltage (Fig. 6a). The optical power of the liquid crystal PBLs can be turned-on and -off by controlling the voltage. Stacking multiple active PBLs can produce 2^N^ depths, where N is the number of PBLs. The drawback of using active PBLs, however, is the limited spectral bandwidth since their diffraction efficiency is usually optimized at a single wavelength. In passive addressing, the depth modulation is achieved through changing the polarization state of input light by a switchable half-wave plate (HWP) (Fig. 6b). The focal length can therefore be switched thanks to the polarization sensitivity of PBLs. Although this approach has a slightly more complicated structure, the overall performance can be better than the active one, because the PBLs made of liquid crystal polymer can be designed to manifest high efficiency within the entire visible spectrum^101,102^.

With the PBL module, multifocal displays can be built using time-multiplexing technique. Zhan et al.^103^ demonstrated a four-depth multifocal display using two actively switchable liquid crystal PBLs (Fig. 6c). The display is synchronized with the PBL module, which lowers the frame rate by the number of depths. Alternatively, multifocal displays can also be achieved by polarization-multiplexing, as demonstrated by Tan et al.^104^. The basic principle is to adjust the polarization state of local pixels so the image content on two focal planes of a PBL can be arbitrarily controlled (Fig. 6d). The advantage of polarization multiplexing is that it does not sacrifice the frame rate, but it can only support two planes because only two orthogonal polarization states are available. Still, it can be combined with time-multiplexing to reduce the frame rate sacrifice by half. Naturally, varifocal displays can also be built with a PBL module. A fast-response 64-depth varifocal module with six PBLs has been demonstrated^105^.

The compact structure of PBL module leads to a natural solution of integrating it with above-mentioned pancake optics. A compact VR headset with dynamic depth modulation to solve VAC is therefore possible in practice. Still, due to the inherent diffractive nature of PBL, the PBL module face the issue of chromatic dispersion of focal length. To compensate for different focal depths for RGB colors may require additional digital corrections in image-rendering.

## Architectures of AR displays

Unlike VR displays with a relatively fixed optical configuration, there exist a vast number of architectures in AR displays. Therefore, instead of following the narrative of tackling different challenges, a more appropriate way to review AR displays is to separately introduce each architecture and discuss its associated engineering challenges. An AR display usually consists of a light engine and an optical combiner. The light engine serves as display image source, while the combiner delivers the displayed images to viewer’s eye and in the meantime transmits the environment light. Some performance parameters like frame rate and power consumption are mainly determined by the light engine. Parameters like FoV, eyebox and MTF are primarily dependent on the combiner optics. Moreover, attributes like image brightness, overall efficiency, and form factor are influenced by both light engine and combiner. In this section, we will firstly discuss the light engine, where the latest advances in micro-LED on chip are reviewed and compared with existing microdisplay systems. Then, we will introduce two main types of combiners: free-space combiner and waveguide combiner.

### Light engine

The light engine determines several essential properties of the AR system like image brightness, power consumption, frame rate, and basic etendue. Several types of microdisplays have been used in AR, including micro-LED, micro-organic-light-emitting-diodes (micro-OLED), liquid-crystal-on-silicon (LCoS), digital micromirror device (DMD), and laser beam scanning (LBS) based on micro-electromechanical system (MEMS). We will firstly describe the working principles of these devices and then analyze their performance. For those who are more interested in final performance parameters than details, Table 1 provides a comprehensive summary.

**Table 1:** Summary of performance parameters of different light engines

	Mechanism	Luminous efficacy	Frame rate	Form factor	Contrast ratio
Micro-LED	Self-emission	5 lm/W (RGB)^130–133^^,a^ 10 lm/W (QD)^114,133^^,a^	State-of-art: N/A Maximum: 1GHz^139^^,b^	Medium (RGB) Small (QD)	>10^6^:1
Micro-OLED	Self-emission	4~8 lm/W^120,122^^,c^	State-of-art: 480 Hz, 8-bit^141^ Maximum: 200 MHz^140^^,b^	Small	>10^6^:1
LCoS	Phase or amplitude modulation	10 lm/W (LED)^127,134,135^ 30 lm/W (laser)^136^	720 Hz, 8-bit (nematic)^125^ 5 kHz, 1-bit (FLC)^125^^,d^	Large (LED) Medium (laser)	(Amplitude) 2000~5000:1
DMD	Amplitude modulation	15 lm/W (LED)^137,138^ 30 lm/W (laser)^136^	30 kHz, 1-bit^d^	Large (LED) Medium (laser)	2000:1
MEMS-LBS	Scanning	40 lm/W^136^	60 Hz, 8-bit^129^	Small	>10^6^:1

^a^May depend on the LED size

^b^Subject to limitation of driving circuits

^c^May face lifetime issue for high brightness

^d^May result in additional power consumption for driving

#### Working principles

**Fig. 7:**
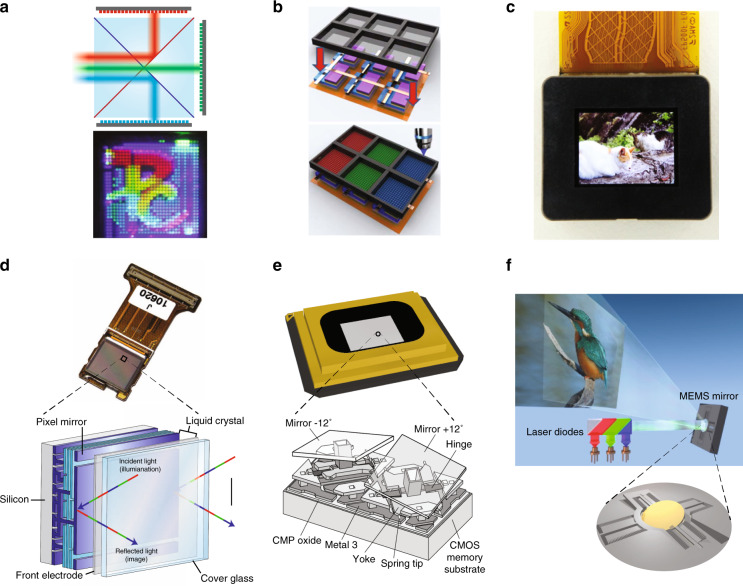
Different types of light engines in AR displays. **a** RGB micro-LED microdisplays combined by a trichroic prism. **b** QD-based micro-LED microdisplay. **c** Micro-OLED display with 4032 PPI. Working principles of **d** LCoS, **e** DMD, and **f** MEMS-LBS display modules. Reprinted from **a** ref. ^106^ with permission from IEEE, **b** ref. ^108^ with permission from Chinese Laser Press, **c** ref. ^121^ with permission from Jon Wiley and Sons, **d** ref. ^124^ with permission from Spring Nature, **e** ref. ^126^ with permission from Springer and **f** ref. ^128^ under the Creative Commons Attribution 4.0 License

Micro-LED and micro-OLED are self-emissive display devices. They are usually more compact than LCoS and DMD because no illumination optics is required. The fundamentally different material systems of LED and OLED lead to different approaches to achieve full-color displays. Due to the “green gap” in LEDs, red LEDs are manufactured on a different semiconductor material from green and blue LEDs. Therefore, how to achieve full-color display in high-resolution density microdisplays is quite a challenge for micro-LEDs. Among several solutions under research are two main approaches. The first is to combine three separate red, green and blue (RGB) micro-LED microdisplay panels^106^. Three single-color micro-LED microdisplays are manufactured separately through flip-chip transfer technology. Then, the projected images from three microdisplay panels are integrated by a trichroic prism (Fig. 7a).

Another solution is to assemble color-conversion materials like quantum dot (QD) on top of blue or ultraviolet (UV) micro-LEDs^107–109^ (Fig. 7b). The quantum dot color filter (QDCF) on top of the micro-LED array is mainly fabricated by inkjet printing or photolithography^110,111^. However, the display performance of color-conversion micro-LED displays is restricted by the low color-conversion efficiency, blue light leakage, and color crosstalk. Extensive efforts have been conducted to improve the QD-micro-LED performance. To boost QD conversion efficiency, structure designs like nanoring^112^ and nanohole^113,114^ have been proposed, which utilize the Förster resonance energy transfer mechanism to transfer excessive excitons in the LED active region to QD. To prevent blue light leakage, methods using color filters or reflectors like distributed Bragg reflector (DBR)^115^ and CLC film^116^ on top of QDCF are proposed. Compared to color filters that absorb blue light, DBR and CLC film help recycle the leaked blue light to further excite QDs. Other methods to achieve full-color micro-LED display like vertically stacked RGB micro-LED array^61,117,118^ and monolithic wavelength tunable nanowire LED^119^ are also under investigation.

Micro-OLED displays can be generally categorized into RGB OLED and white OLED (WOLED). RGB OLED displays have separate sub-pixel structures and optical cavities, which resonate at the desirable wavelength in RGB channels, respectively. To deposit organic materials onto the separated RGB sub-pixels, a fine metal mask (FMM) that defines the deposition area is required. However, high-resolution RGB OLED microdisplays still face challenges due to the shadow effect during the deposition process through FMM. In order to break the limitation, a silicon nitride film with small shadow has been proposed as a mask for high-resolution deposition above 2000 PPI (9.3 µm)^120^.

WOLED displays use color filters to generate color images. Without the process of depositing patterned organic materials, a high-resolution density up to 4000 PPI has been achieved^121^ (Fig. 7c). However, compared to RGB OLED, the color filters in WOLED absorb about 70% of the emitted light, which limits the maximum brightness of the microdisplay. To improve the efficiency and peak brightness of WOLED microdisplays, in 2019 Sony proposed to apply newly designed cathodes (InZnO) and microlens arrays on OLED microdisplays, which increased the peak brightness from 1600 nits to 5000 nits^120^. In addition, OLEDWORKs has proposed a multi-stacked OLED^122^ with optimized microcavities whose emission spectra match the transmission bands of the color filters. The multi-stacked OLED shows a higher luminous efficiency (cd/A), but also requires a higher driving voltage. Recently, by using meta-mirrors as bottom reflective anodes, patterned microcavities with more than 10,000 PPI have been obtained^123^. The high-resolution meta-mirrors generate different reflection phases in the RGB sub-pixels to achieve desirable resonant wavelengths. The narrow emission spectra from the microcavity help to reduce the loss from color filters or even eliminate the need of color filters.

LCoS and DMD are light-modulating displays that generate images by controlling the reflection of each pixel. For LCoS, the light modulation is achieved by manipulating the polarization state of output light through independently controlling the liquid crystal reorientation in each pixel^124,125^ (Fig. 7d). Both phase-only and amplitude modulators have been employed. DMD is an amplitude modulation device. The modulation is achieved through controlling the tilt angle of bi-stable micromirrors^126^ (Fig. 7e). To generate an image, both LCoS and DMD rely on the light illumination systems, with LED or laser as light source. For LCoS, the generation of color image can be realized either by RGB color filters on LCoS (with white LEDs) or color-sequential addressing (with RGB LEDs or lasers). However, LCoS requires a linearly polarized light source. For an unpolarized LED light source, usually, a polarization recycling system^127^ is implemented to improve the optical efficiency. For a single-panel DMD, the color image is mainly obtained through color-sequential addressing. In addition, DMD does not require a polarized light so that it generally exhibits a higher efficiency than LCoS if an unpolarized light source is employed.

MEMS-based LBS^128,129^ utilizes micromirrors to directly scan RGB laser beams to form two-dimensional (2D) images (Fig. 7f). Different gray levels are achieved by pulse width modulation (PWM) of the employed laser diodes. In practice, 2D scanning can be achieved either through a 2D scanning mirror or two 1D scanning mirrors with an additional focusing lens after the first mirror. The small size of MEMS mirror offers a very attractive form factor. At the same time, the output image has a large depth-of-focus (DoF), which is ideal for projection displays. One shortcoming, though, is that the small system etendue often hinders its applications in some traditional display systems.

#### Comparison of light engine performance

There are several important parameters for a light engine, including image resolution, brightness, frame rate, contrast ratio, and form factor. The resolution requirement (>2K) is similar for all types of light engines. The improvement of resolution is usually accomplished through the manufacturing process. Thus, here we shall focus on other three parameters.

Image brightness usually refers to the measured luminance of a light-emitting object. This measurement, however, may not be accurate for a light engine as the light from engine only forms an intermediate image, which is not directly viewed by the user. On the other hand, to solely focus on the brightness of a light engine could be misleading for a wearable display system like AR. Nowadays, data projectors with thousands of lumens are available. But the power consumption is too high for a battery-powered wearable AR display. Therefore, a more appropriate way to evaluate a light engine’s brightness is to use luminous efficacy (lm/W) measured by dividing the final output luminous flux (lm) by the input electric power (W). For a self-emissive device like micro-LED or micro-OLED, the luminous efficacy is directly determined by the device itself. However, for LCoS and DMD, the overall luminous efficacy should take into consideration the light source luminous efficacy, the efficiency of illumination optics, and the efficiency of the employed spatial light modulator (SLM). For a MEMS LBS engine, the efficiency of MEMS mirror can be considered as unity so that the luminous efficacy basically equals to that of the employed laser sources.

As mentioned earlier, each light engine has a different scheme for generating color images. Therefore, we separately list luminous efficacy of each scheme for a more inclusive comparison. For micro-LEDs, the situation is more complicated because the EQE depends on the chip size. Based on previous studies^130–133^, we separately calculate the luminous efficacy for RGB micro-LEDs with chip size ≈ 20 µm. For the scheme of direct combination of RGB micro-LEDs, the luminous efficacy is around 5 lm/W. For QD-conversion with blue micro-LEDs, the luminous efficacy is around 10 lm/W with the assumption of 100% color conversion efficiency, which has been demonstrated using structure engineering^114^. For micro-OLEDs, the calculated luminous efficacy is about 4–8 lm/W^120,122^. However, the lifetime and EQE of blue OLED materials depend on the driving current. To continuously display an image with brightness higher than 10,000 nits may dramatically shorten the device lifetime. The reason we compare the light engine at 10,000 nits is that it is highly desirable to obtain 1000 nits for the displayed image in order to keep ACR>3:1 with a typical AR combiner whose optical efficiency is lower than 10%.

For an LCoS engine using a white LED as light source, the typical optical efficiency of the whole engine is around 10%^127,134^. Then the engine luminous efficacy is estimated to be 12 lm/W with a 120 lm/W white LED source. For a color sequential LCoS using RGB LEDs, the absorption loss from color filters is eliminated, but the luminous efficacy of RGB LED source is also decreased to about 30 lm/W due to lower efficiency of red and green LEDs and higher driving current^135^. Therefore, the final luminous efficacy of the color sequential LCoS engine is also around 10 lm/W. If RGB linearly polarized lasers are employed instead of LEDs, then the LCoS engine efficiency can be quite high due to the high degree of collimation. The luminous efficacy of RGB laser source is around 40 lm/W^136^. Therefore, the laser-based LCoS engine is estimated to have a luminous efficacy of 32 lm/W, assuming the engine optical efficiency is 80%. For a DMD engine with RGB LEDs as light source, the optical efficiency is around 50%^137,138^, which leads to a luminous efficacy of 15 lm/W. By switching to laser light sources, the situation is similar to LCoS, with the luminous efficacy of about 32 lm/W. Finally, for MEMS-based LBS engine, there is basically no loss from the optics so that the final luminous efficacy is 40 lm/W. Detailed calculations of luminous efficacy can be found in Supplementary Information.

Another aspect of a light engine is the frame rate, which determines the volume of information it can deliver in a unit time. A high volume of information is vital for the construction of a 3D light field to solve the VAC issue. For micro-LEDs, the device response time is around several nanoseconds, which allows for visible light communication with bandwidth up to 1.5 Gbit/s^139^. For an OLED microdisplay, a fast OLED with ~200 MHz bandwidth has been demonstrated^140^. Therefore, the limitation of frame rate is on the driving circuits for both micro-LED and OLED. Another fact concerning driving circuit is the tradeoff between resolution and frame rate as a higher resolution panel means more scanning lines in each frame. So far, an OLED display with 480 Hz frame rate has been demonstrated^141^. For an LCoS, the frame rate is mainly limited by the LC response time. Depending on the LC material used, the response time is around 1 ms for nematic LC or 200 µs for ferroelectric LC (FLC)^125^. Nematic LC allows analog driving, which accommodates gray levels, typically with 8-bit depth. FLC is bistable so that PWM is used to generate gray levels. DMD is also a binary device. The frame rate can reach 30 kHz, which is mainly constrained by the response time of micromirrors. For MEMS-based LBS, the frame rate is limited by the scanning frequency of MEMS mirrors. A frame rate of 60 Hz with around 1 K resolution already requires a resonance frequency of around 50 kHz, with a Q-factor up to 145,000^128^. A higher frame rate or resolution requires a higher Q-factor and larger laser modulation bandwidth, which may be challenging.

Form factor is another crucial aspect for the light engines of near-eye displays. For self-emissive displays, both micro-OLEDs and QD-based micro-LEDs can achieve full color with a single panel. Thus, they are quite compact. A micro-LED display with separate RGB panels naturally have a larger form factor. In applications requiring direct-view full-color panel, the extra combining optics may also increase the volume. It needs to be pointed out, however, that the combing optics may not be necessary for some applications like waveguide displays, because the EPE process results in system’s insensitivity to the spatial positions of input RGB images. Therefore, the form factor of using three RGB micro-LED panels is medium. For LCoS and DMD with RGB LEDs as light source, the form factor would be larger due to the illumination optics. Still, if a lower luminous efficacy can be accepted, then a smaller form factor can be achieved by using a simpler optics^142^. If RGB lasers are used, the collimation optics can be eliminated, which greatly reduces the form factor^143^. For MEMS-LBS, the form factor can be extremely compact due to the tiny size of MEMS mirror and laser module.

Finally, contrast ratio (CR) also plays an important role affecting the observed images^8^. Micro-LEDs and micro-OLEDs are self-emissive so that their CR can be >10^6^:1. For a laser beam scanner, its CR can also achieve 10^6^:1 because the laser can be turned off completely at dark state. On the other hand, LCoS and DMD are reflective displays, and their CR is around 2000:1 to 5000:1^144,145^. It is worth pointing out that the CR of a display engine plays a significant role only in the dark ambient. As the ambient brightness increases, the ACR is mainly governed by the display’s peak brightness, as previously discussed.

The performance parameters of different light engines are summarized in Table 1. Micro-LEDs and micro-OLEDs have similar levels of luminous efficacy. But micro-OLEDs still face the burn-in and lifetime issue when driving at a high current, which hinders its use for a high-brightness image source to some extent. Micro-LEDs are still under active development and the improvement on luminous efficacy from maturing fabrication process could be expected. Both devices have nanosecond response time and can potentially achieve a high frame rate with a well-designed integrated circuit. The frame rate of the driving circuit ultimately determines the motion picture response time^146^. Their self-emissive feature also leads to a small form factor and high contrast ratio. LCoS and DMD engines have similar performance of luminous efficacy, form factor, and contrast ratio. In terms of light modulation, DMD can provide a higher 1-bit frame rate, while LCoS can offer both phase and amplitude modulations. MEMS-based LBS exhibits the highest luminous efficacy so far. It also exhibits an excellent form factor and contrast ratio, but the presently demonstrated 60-Hz frame rate (limited by the MEMS mirrors) could cause image flickering.

### Free-space combiners

The term ‘free-space’ generally refers to the case when light is freely propagating in space, as opposed to a waveguide that traps light into TIRs. Regarding the combiner, it can be a partial mirror, as commonly used in AR systems based on traditional geometric optics. Alternatively, the combiner can also be a reflective HOE. The strong chromatic dispersion of HOE necessitates the use of a laser source, which usually leads to a Maxwellian-type system.

#### Traditional geometric designs

**Fig. 8:**
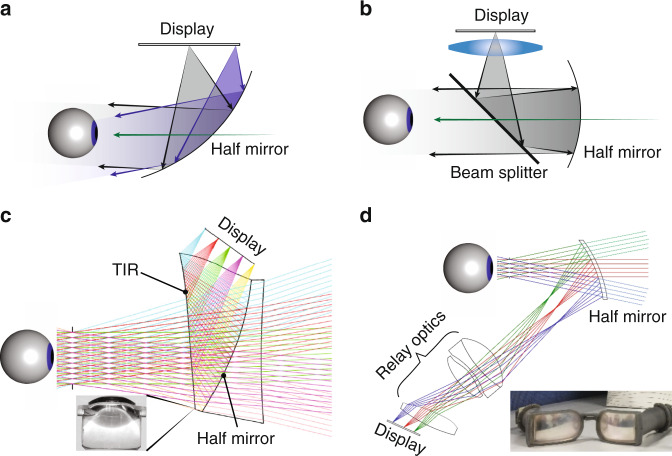
AR combiners based on geometric optical designs. **a** Single freeform surface as the combiner. **b** Birdbath optics with a beam splitter and a half mirror. **c** Freeform TIR prism with a compensator. **d** Relay optics with a half mirror. Adapted from **c** ref. ^149^ with permission from OSA Publishing and **d** ref. ^151^ with permission from OSA Publishing

Several systems based on geometric optics are illustrated in Fig. 8. The simplest design uses a single freeform half-mirror^6,147^ to directly collimate the displayed images to the viewer’s eye (Fig. 8a). This design can achieve a large FoV (up to 90°)^147^, but the limited design freedom with a single freeform surface leads to image distortions, also called pupil swim^6^. The placement of half-mirror also results in a relatively bulky form factor. Another design using so-called birdbath optics^6,148^ is shown in Fig. 8b. Compared to the single-combiner design, birdbath design has an extra optics on the display side, which provides space for aberration correction. The integration of beam splitter provides a folded optical path, which reduces the form factor to some extent. Another way to fold optical path is to use a TIR-prism. Cheng et al.^149^ designed a freeform TIR-prism combiner (Fig. 8c) offering a diagonal FoV of 54° and exit pupil diameter of 8 mm. All the surfaces are freeform, which offer an excellent image quality. To cancel the optical power for the transmitted environmental light, a compensator is added to the TIR prism. The whole system has a well-balanced performance between FoV, eyebox, and form factor. To release the space in front of viewer’s eye, relay optics can be used to form an intermediate image near the combiner^150,151^, as illustrated in Fig. 8d. Although the design offers more optical surfaces for aberration correction, the extra lenses also add to system weight and form factor.

Regarding the approaches to solve the VAC issue, the most straightforward way is to integrate a tunable lens into the optical path, like a liquid lens^152^ or Alvarez lens^99^, to form a varifocal system. Alternatively, integral imaging^153,154^ can also be used, by replacing the original display panel with the central depth plane of an integral imaging module. The integral imaging can also be combined with varifocal approach to overcome the tradeoff between resolution and depth of field (DoF)^155–157^. However, the inherent tradeoff between resolution and view number still exists in this case.

Overall, AR displays based on traditional geometric optics have a relatively simple design with a decent FoV (~60°) and eyebox (8 mm)^158^. They also exhibit a reasonable efficiency. To measure the efficiency of an AR combiner, an appropriate measure is to divide the output luminance (unit: nit) by the input luminous flux (unit: lm), which we note as combiner efficiency. For a fixed input luminous flux, the output luminance, or image brightness, is related to the FoV and exit pupil of the combiner system. If we assume no light waste of the combiner system, then the maximum combiner efficiency for a typical diagonal FoV of 60° and exit pupil (10 mm square) is around 17,000 nit/lm (Eq. S2). To estimate the combiner efficiency of geometric combiners, we assume 50% of half-mirror transmittance and the efficiency of other optics to be 50%. Then the final combiner efficiency is about 4200 nit/lm, which is a high value in comparison with waveguide combiners. Nonetheless, to further shrink the system size or improve system performance ultimately encounters the etendue conservation issue. In addition, AR systems with traditional geometric optics is hard to achieve a configuration resembling normal flat glasses because the half-mirror has to be tilted to some extent.

#### Maxwellian-type systems

**Fig. 9:**
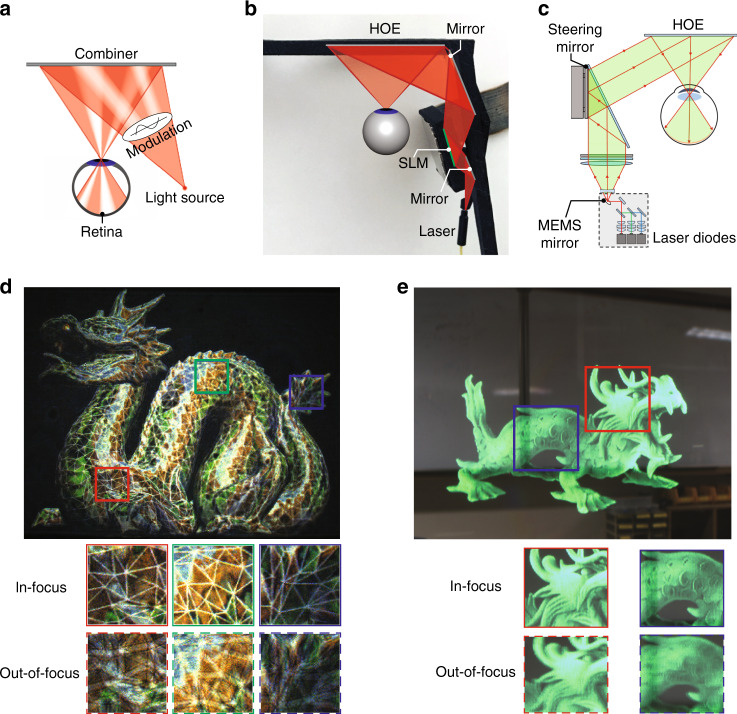
Maxwellian-type AR Combiners. **a** Schematic of the working principle of Maxwellian displays. Maxwellian displays based on **b** SLM and laser diode light source and **c** MEMS-LBS with a steering mirror as additional modulation method. Generation of depth cues by **d** computational digital holography and **e** scanning of steering mirror to produce multiple views. Adapted from **b, d** ref. ^143^ and **c, e** ref. ^167^ under the Creative Commons Attribution 4.0 License

The Maxwellian view, proposed by James Clerk Maxwell (1860), refers to imaging a point light source in the eye pupil^159^. If the light beam is modulated in the imaging process, a corresponding image can be formed on the retina (Fig. 9a). Because the point source is much smaller than the eye pupil, the image is always-in-focus on the retina irrespective of the eye lens’ focus. For applications in AR display, the point source is usually a laser with narrow angular and spectral bandwidths. LED light sources can also build a Maxwellian system, by adding an angular filtering module^160^. Regarding the combiner, although in theory a half-mirror can also be used, HOEs are generally preferred because they offer the off-axis configuration that places combiner in a similar position like eyeglasses. In addition, HOEs have a lower reflection of environment light, which provides a more natural appearance of the user behind the display.

To modulate the light, a SLM like LCoS or DMD can be placed in the light path, as shown in Fig. 9b. Alternatively, LBS system can also be used (Fig. 9c), where the intensity modulation occurs in the laser diode itself. Besides the operation in a normal Maxwellian-view, both implementations offer additional degrees of freedom for light modulation.

For a SLM-based system, there are several options to arrange the SLM pixels^143,161^. Maimone et al.^143^ demonstrated a Maxwellian AR display with two modes to offer a large-DoF Maxwellian-view, or a holographic view (Fig. 9d), which is often referred as computer-generated holography (CGH)^162^. To show an always-in-focus image with a large DoF, the image can be directly displayed on an amplitude SLM, or using amplitude encoding for a phase-only SLM^163^. Alternatively, if a 3D scene with correct depth cues is to be presented, then optimization algorithms for CGH can be used to generate a hologram for the SLM. The generated holographic image exhibits the natural focus-and-blur effect like a real 3D object (Fig. 9d). To better understand this feature, we need to again exploit the concept of etendue. The laser light source can be considered to have a very small etendue due to its excellent collimation. Therefore, the system etendue is provided by the SLM. The micron-sized pixel-pitch of SLM offers a certain maximum diffraction angle, which, multiplied by the SLM size, equals system etendue. By varying the display content on SLM, the final exit pupil size can be changed accordingly. In the case of a large-DoF Maxwellian view, the exit pupil size is small, accompanied by a large FoV. For the holographic display mode, the reduced DoF requires a larger exit pupil with dimension close to the eye pupil. But the FoV is reduced accordingly due to etendue conservation. Another commonly concerned issue with CGH is the computation time. To achieve a real-time CGH rendering flow with an excellent image quality is quite a challenge. Fortunately, with recent advances in algorithm^164^ and the introduction of convolutional neural network (CNN)^165,166^, this issue is gradually solved with an encouraging pace. Lately, Liang et al.^166^ demonstrated a real-time CGH synthesis pipeline with a high image quality. The pipeline comprises an efficient CNN model to generate a complex hologram from a 3D scene and an improved encoding algorithm to convert the complex hologram to a phase-only one. An impressive frame rate of 60 Hz has been achieved on a desktop computing unit.

For LBS-based system, the additional modulation can be achieved by integrating a steering module, as demonstrated by Jang et al.^167^. The steering mirror can shift the focal point (viewpoint) within the eye pupil, therefore effectively expanding the system etendue. When the steering process is fast and the image content is updated simultaneously, correct 3D cues can be generated, as shown in Fig. 9e. However, there exists a tradeoff between the number of viewpoint and the final image frame rate, because the total frames are equally divided into each viewpoint. To boost the frame rate of MEMS-LBS systems by the number of views (e.g., 3 by 3) may be challenging.

Maxwellian-type systems offer several advantages. The system efficiency is usually very high because nearly all the light is delivered into viewer’s eye. The system FoV is determined by the *f*/# of combiner and a large FoV (~80° in horizontal) can be achieved^143^. The issue of VAC can be mitigated with an infinite-DoF image that deprives accommodation cue, or completely solved by generating a true-3D scene as discussed above. Despite these advantages, one major weakness of Maxwellian-type system is the tiny exit pupil, or eyebox. A small deviation of eye pupil location from the viewpoint results in the complete disappearance of the image. Therefore, to expand eyebox is considered as one of the most important challenges in Maxwellian-type systems.

#### Pupil duplication and steering

Methods to expand eyebox can be generally categorized into pupil duplication^168–172^ and pupil steering^9,13,167,173^. Pupil duplication simply generates multiple viewpoints to cover a large area. In contrast, pupil steering dynamically shifts the viewpoint position, depending on the pupil location. Before reviewing detailed implementations of these two methods, it is worth discussing some of their general features. The multiple viewpoints in pupil duplication usually mean to equally divide the total light intensity. In each time frame, however, it is preferable that only one viewpoint enters the user’s eye pupil to avoid ghost image. This requirement, therefore, results in a reduced total light efficiency, while also conditioning the viewpoint separation to be larger than the pupil diameter. In addition, the separation should not be too large to avoid gap between viewpoints. Considering that human pupil diameter changes in response to environment illuminance, the design of viewpoint separation needs special attention. Pupil steering, on the other hand, only produces one viewpoint at each time frame. It is therefore more light-efficient and free from ghost images. But to determine the viewpoint position requires the information of eye pupil location, which demands a real-time eye-tracking module^9^. Another observation is that pupil steering can accommodate multiple viewpoints by its nature. Therefore, a pupil steering system can often be easily converted to a pupil duplication system by simultaneously generating available viewpoints.

**Fig. 10:**
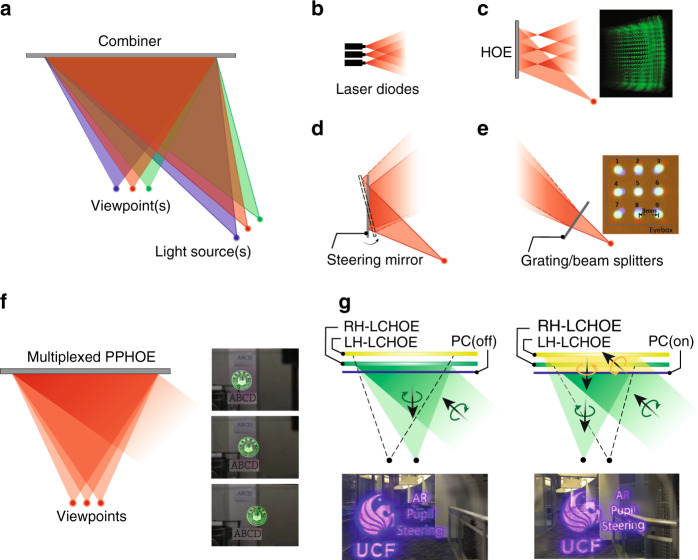
Methods of pupil duplication and pupil steering. **a** Schematic of duplicating (or shift) viewpoint by modulation of incident light. Light modulation by **b** multiple laser diodes, **c** HOE lens array, **d** steering mirror and **e** grating or beam splitters. **f** Pupil duplication with multiplexed PPHOE. **g** Pupil steering with LCHOE. Reproduced from **c** ref. ^168^ under the Creative Commons Attribution 4.0 License, **e** ref. ^169^ with permission from OSA Publishing, **f** ref. ^171^ with permission from OSA Publishing and **g** ref. ^173^ with permission from OSA Publishing

To generate multiple viewpoints, one can focus on modulating the incident light or the combiner. Recall that viewpoint is the image of light source. To duplicate or shift light source can achieve pupil duplication or steering accordingly, as illustrated in Fig. 10a. Several schemes of light modulation are depicted in Fig. 10b–e. An array of light sources can be generated with multiple laser diodes (Fig. 10b). To turn on all or one of the sources achieves pupil duplication or steering. A light source array can also be produced by projecting light on an array-type PPHOE^168^ (Fig. 10c). Apart from direct adjustment of light sources, modulating light on the path can also effectively steer/duplicate the light sources. Using a mechanical steering mirror, the beam can be deflected^167^ (Fig. 10d), which equals to shifting the light source position. Other devices like a grating or beam splitter can also serve as ray deflector/splitter^170,171^ (Fig. 10e).

Nonetheless, one problem of the light source duplication/shifting methods for pupil duplication/steering is that the aberrations in peripheral viewpoints are often serious^168,173^. The HOE combiner is usually recorded at one incident angle. For other incident angles with large deviations, considerable aberrations will occur, especially in the scenario of off-axis configuration. To solve this problem, the modulation can be focused on the combiner instead. While the mechanical shifting of combiner^9^ can achieve continuous pupil steering, its integration into AR display with a small factor remains a challenge. Alternatively, the versatile functions of HOE offer possible solutions for combiner modulation. Kim and Park^169^ demonstrated a pupil duplication system with multiplexed PPHOE (Fig. 10f). Wavefronts of several viewpoints can be recorded into one PPHOE sample. Three viewpoints with a separation of 3 mm were achieved. However, a slight degree of ghost image and gap can be observed in the viewpoint transition. For a PPHOE to achieve pupil steering, the multiplexed PPHOE needs to record different focal points with different incident angles. If each hologram has no angular crosstalk, then with an additional device to change the light incident angle, the viewpoint can be steered. Alternatively, Xiong et al.^173^ demonstrated a pupil steering system with LCHOEs in a simpler configuration (Fig. 10g). The polarization-sensitive nature of LCHOE enables the controlling of which LCHOE to function with a polarization converter (PC). When the PC is off, the incident RCP light is focused by the right-handed LCHOE. When the PC is turned on, the RCP light is firstly converted to LCP light and passes through the right-handed LCHOE. Then it is focused by the left-handed LCHOE into another viewpoint. To add more viewpoints requires stacking more pairs of PC and LCHOE, which can be achieved in a compact manner with thin glass substrates. In addition, to realize pupil duplication only requires the stacking of multiple low-efficiency LCHOEs. For both PPHOEs and LCHOEs, because the hologram for each viewpoint is recorded independently, the aberrations can be eliminated.

Regarding the system performance, in theory the FoV is not limited and can reach a large value, such as 80° in horizontal direction^143^. The definition of eyebox is different from traditional imaging systems. For a single viewpoint, it has the same size as the eye pupil diameter. But due to the viewpoint steering/duplication capability, the total system eyebox can be expanded accordingly. The combiner efficiency for pupil steering systems can reach 47,000 nit/lm for a FoV of 80° by 80° and pupil diameter of 4 mm (Eq. S2). At such a high brightness level, eye safety could be a concern^174^. For a pupil duplication system, the combiner efficiency is decreased by the number of viewpoints. With a 4-by-4 viewpoint array, it can still reach 3000 nit/lm. Despite the potential gain of pupil duplication/steering, when considering the rotation of eyeball, the situation becomes much more complicated^175^. A perfect pupil steering system requires a 5D steering, which proposes a challenge for practical implementation.

#### Pin-light systems

**Fig. 11:**
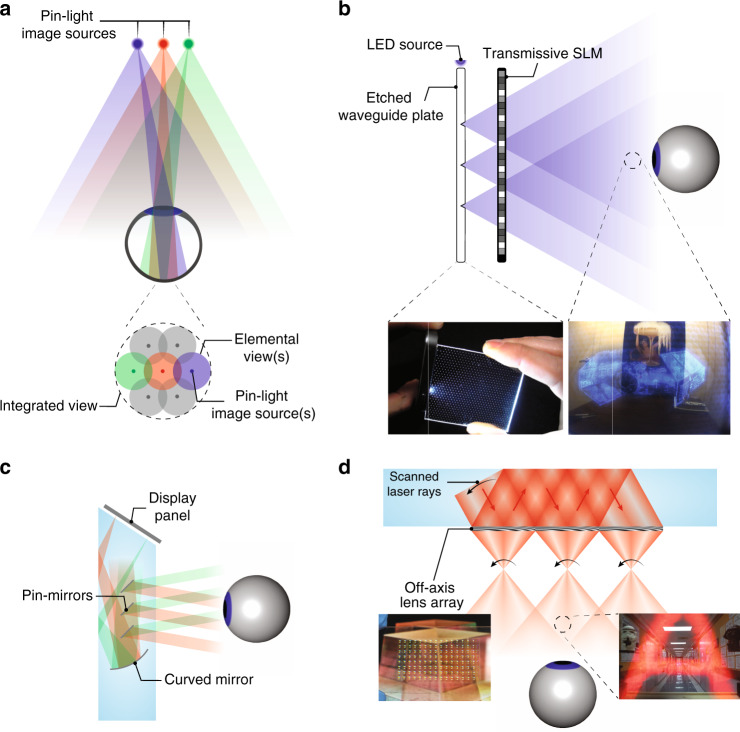
Illustration of pin-light displays. **a** Schematic drawing of the working principle of pin-light display. **b** Pin-light display utilizing a pin-light source and a transmissive SLM. **c** An example of pin-mirror display with a birdbath optics. **d** SWD system with LBS image source and off-axis lens array. Reprinted from **b** ref. ^176^ under the Creative Commons Attribution 4.0 License and **d** ref. ^180^ with permission from OSA Publishing

Recently, another type of display in close relation with Maxwellian view called pin-light display^148,176^ has been proposed. The general working principle of pin-light display is illustrated in Fig. 11a. Each pin-light source is a Maxwellian view with a large DoF. When the eye pupil is no longer placed near the source point as in Maxwellian view, each image source can only form an elemental view with a small FoV on retina. However, if the image source array is arranged in a proper form, the elemental views can be integrated together to form a large FoV. According to the specific optical architectures, pin-light display can take different forms of implementation. In the initial feasibility demonstration, Maimone et al.^176^ used a side-lit waveguide plate as the point light source (Fig. 11b). The light inside the waveguide plate is extracted by the etched divots, forming a pin-light source array. A transmissive SLM (LCD) is placed behind the waveguide plate to modulate the light intensity and form the image. The display has an impressive FoV of 110° thanks to the large scattering angle range. However, the direct placement of LCD before the eye brings issues of insufficient resolution density and diffraction of background light.

To avoid these issues, architectures using pin-mirrors^177–179^ are proposed. In these systems, the final combiner is an array of tiny mirrors^178,179^ or gratings^177^, in contrast to their counterparts using large-area combiners. An exemplary system with birdbath design is depicted in Fig. 11c. In this case, the pin-mirrors replace the original beam-splitter in the birdbath and can thus shrink the system volume, while at the same time providing large DoF pin-light images. Nonetheless, such a system may still face the etendue conservation issue. Meanwhile, the size of pin-mirror cannot be too small in order to prevent degradation of resolution density due to diffraction. Therefore, its influence on the see-through background should also be considered in the system design.

To overcome the etendue conservation and improve see-through quality, Xiong et al.^180^ proposed another type of pin-light system exploiting the etendue expansion property of waveguide, which is also referred as scanning waveguide display (SWD). As illustrated in Fig. 11d, the system uses an LBS as the image source. The collimated scanned laser rays are trapped in the waveguide and encounter an array of off-axis lenses. Upon each encounter, the lens out-couples the laser rays and forms a pin-light source. SWD has the merits of good see-through quality and large etendue. A large FoV of 100° was demonstrated with the help of an ultra-low *f*/# lens array based on LCHOE. However, some issues like insufficient image resolution density and image non-uniformity remain to be overcome. To further improve the system may require optimization of Gaussian beam profile and additional EPE module^180^.

Overall, pin-light systems inherit the large DoF from Maxwellian view. With adequate number of pin-light sources, the FoV and eyebox can be expanded accordingly. Nonetheless, despite different forms of implementation, a common issue of pin-light system is the image uniformity. The overlapped region of elemental views has a higher light intensity than the non-overlapped region, which becomes even more complicated considering the dynamic change of pupil size. In theory, the displayed image can be pre-processed to compensate for the optical non-uniformity. But that would require knowledge of precise pupil location (and possibly size) and therefore an accurate eye-tracking module^176^. Regarding the system performance, pin-mirror systems modified from other free-space systems generally shares similar FoV and eyebox with original systems. The combiner efficiency may be lower due to the small size of pin-mirrors. SWD, on the other hand, shares the large FoV and DoF with Maxwellian view, and large eyebox with waveguide combiners. The combiner efficiency may also be lower due to the EPE process.

### Waveguide combiner

Besides free-space combiners, another common architecture in AR displays is waveguide combiner. The term ‘waveguide’ indicates the light is trapped in a substrate by the TIR process. One distinctive feature of a waveguide combiner is the EPE process that effectively enlarges the system etendue. In the EPE process, a portion of the trapped light is repeatedly coupled out of the waveguide in each TIR. The effective eyebox is therefore enlarged. According to the features of couplers, we divide the waveguide combiners into two types: diffractive and achromatic, as described in the followings.

#### Diffractive waveguides

As the name implies, diffractive-type waveguides use diffractive elements as couplers. The in-coupler is usually a diffractive grating and the out-coupler in most cases is also a grating with the same period as the in-coupler, but it can also be an off-axis lens with a small curvature to generate image with finite depth. Three major diffractive couplers have been developed: SRGs, photopolymer gratings (PPGs), and liquid crystal gratings (grating-type LCHOE; also known as polarization volume gratings (PVGs)). Some general protocols for coupler design are that the in-coupler should have a relatively high efficiency and the out-coupler should have a uniform light output. A uniform light output usually requires a low-efficiency coupler, with extra degrees of freedom for local modulation of coupling efficiency. Both in-coupler and out-coupler should have an adequate angular bandwidth to accommodate a reasonable FoV. In addition, the out-coupler should also be optimized to avoid undesired diffractions, including the outward diffraction of TIR light and diffraction of environment light into user’s eyes, which are referred as light leakage and rainbow. Suppression of these unwanted diffractions should also be considered in the optimization process of waveguide design, along with performance parameters like efficiency and uniformity.

**Fig. 12:**
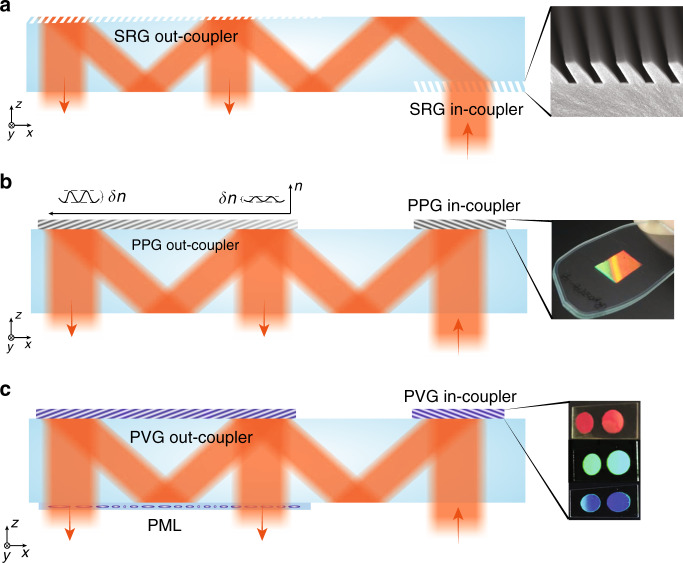
Working principles of diffractive waveguide combiners. Schematics of waveguide combiners based on **a** SRGs, **b** PPGs and **c** PVGs. Reprinted from **a** ref. ^85^ with permission from OSA Publishing, **b** ref. ^185^ with permission from John Wiley and Sons and **c** ref. ^189^ with permission from OSA Publishing

The basic working principles of diffractive waveguide-based AR systems are illustrated in Fig. 12. For the SRG-based waveguides^6,8^ (Fig. 12a), the in-coupler can be a transmissive-type or a reflective-type^181,182^. The grating geometry can be optimized for coupling efficiency with a large degree of freedom^183^. For the out-coupler, a reflective SRG with a large slant angle to suppress the transmission orders is preferred^184^. In addition, a uniform light output usually requires a gradient efficiency distribution in order to compensate for the decreased light intensity in the out-coupling process. This can be achieved by varying the local grating configurations like height and duty cycle^6^. For the PPG-based waveguides^185^ (Fig. 12b), the small angular bandwidth of a high-efficiency transmissive PPG prohibits its use as in-coupler. Therefore, both in-coupler and out-coupler are usually reflective types. The gradient efficiency can be achieved by space-variant exposure to control the local index modulation^186^ or local Bragg slant angle variation through freeform exposure^19^. Due to the relatively small angular bandwidth of PPG, to achieve a decent FoV usually requires stacking two^187^ or three^188^ PPGs together for a single color. The PVG-based waveguides^189^ (Fig. 12c) also prefer reflective PVGs as in-couplers because the transmissive PVGs are much more difficult to fabricate due to the LC alignment issue. In addition, the angular bandwidth of transmissive PVGs in Bragg regime is also not large enough to support a decent FoV^29^. For the out-coupler, the angular bandwidth of a single reflective PVG can usually support a reasonable FoV. To obtain a uniform light output, a polarization management layer^190^ consisting of a LC layer with spatially variant orientations can be utilized. It offers an additional degree of freedom to control the polarization state of the TIR light. The diffraction efficiency can therefore be locally controlled due to the strong polarization sensitivity of PVG.

**Fig. 13:**
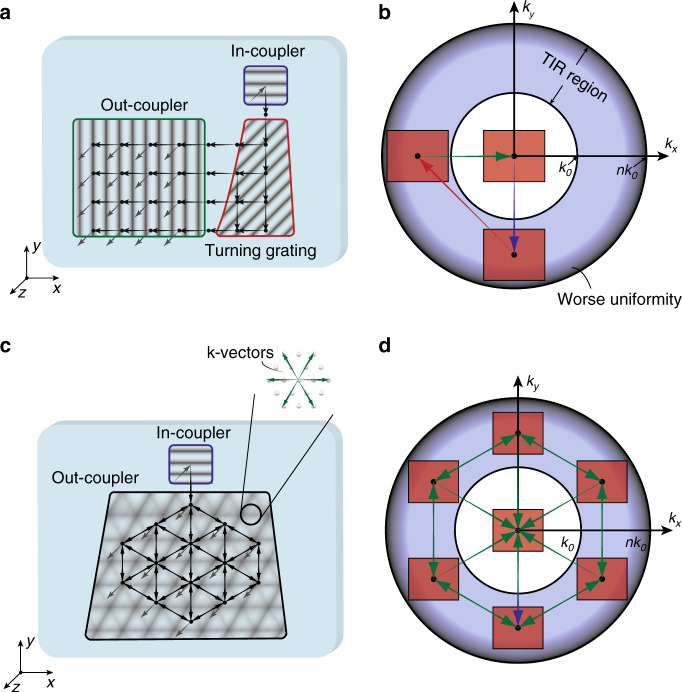
Schemes for 2D EPE. **a** Schematic of 2D EPE based on two consecutive 1D EPEs. Gray/black arrows indicate light in air/TIR. Black dots denote TIRs. **b** k-diagram of the two-1D-EPE scheme. **c** Schematic of 2D EPE with a 2D hexagonal grating **d** k-diagram of the 2D-grating scheme

The above discussion describes the basic working principle of 1D EPE. Nonetheless, for the 1D EPE to produce a large eyebox, the exit pupil in the unexpanded direction of the original image should be large. This proposes design challenges in light engines. Therefore, a 2D EPE is favored for practical applications. To extend EPE in two dimensions, two consecutive 1D EPEs can be used^191^, as depicted in Fig. 13a. The first 1D EPE occurs in the turning grating, where the light is duplicated in *y* direction and then turned into *x* direction. Then the light rays encounter the out-coupler and are expanded in *x* direction. To better understand the 2D EPE process, the *k*-vector diagram (Fig. 13b) can be used. For the light propagating in air with wavenumber *k*_*0*_, its possible *k*-values in *x* and *y* directions (*k*_*x*_ and *k*_*y*_) fall within the circle with radius *k*_*0*_. When the light is trapped into TIR, *k*_*x*_ and *k*_*y*_ are outside the circle with radius *k*_*0*_ and inside the circle with radius *nk*_*0*_, where *n* is the refractive index of the substrate. *k*_*x*_ and *k*_*y*_ stay unchanged in the TIR process and are only changed in each diffraction process. The central red box in Fig. 13b indicates the possible *k* values within the system FoV. After the in-coupler, the *k* values are added by the grating *k*-vector, shifting the *k* values into TIR region. The turning grating then applies another *k*-vector and shifts the *k* values to near *x*-axis. Finally, the *k* values are shifted by the out-coupler and return to the free propagation region in air. One observation is that the size of red box is mostly limited by the width of TIR band. To accommodate a larger FoV, the outer boundary of TIR band needs to be expanded, which amounts to increasing waveguide refractive index. Another important fact is that when *k*_*x*_ and *k*_*y*_ are near the outer boundary, the uniformity of output light becomes worse. This is because the light propagation angle is near 90° in the waveguide. The spatial distance between two consecutive TIRs becomes so large that the out-coupled beams are spatially separated to an unacceptable degree. The range of possible *k* values for practical applications is therefore further shrunk due to this fact.

Aside from two consecutive 1D EPEs, the 2D EPE can also be directly implemented with a 2D grating^192^. An example using a hexagonal grating is depicted in Fig. 13c. The hexagonal grating can provide *k*-vectors in six directions. In the *k*-diagram (Fig. 13d), after the in-coupling, the *k* values are distributed into six regions due to multiple diffractions. The out-coupling occurs simultaneously with pupil expansion. Besides a concise out-coupler configuration, the 2D EPE scheme offers more degrees of design freedom than two 1D EPEs because the local grating parameters can be adjusted in a 2D manner. The higher design freedom has the potential to reach a better output light uniformity, but at the cost of a higher computation demand for optimization. Furthermore, the unslanted grating geometry usually leads to a large light leakage and possibly low efficiency. Adding slant to the geometry helps alleviate the issue, but the associated fabrication may be more challenging.

Finally, we discuss the generation of full-color images. One important issue to clarify is that although diffractive gratings are used here, the final image generally has no color dispersion even if we use a broadband light source like LED. This can be easily understood in the 1D EPE scheme. The in-coupler and out-coupler have opposite *k*-vectors, which cancels the color dispersion for each other. In the 2D EPE schemes, the *k*-vectors always form a closed loop from in-coupled light to out-coupled light, thus, the color dispersion also vanishes likewise. The issue of using a single waveguide for full-color images actually exists in the consideration of FoV and light uniformity. The breakup of propagation angles for different colors results in varied out-coupling situations for each color. To be more specific, if the red and the blue channels use the same in-coupler, the propagating angle for the red light is larger than that of the blue light. The red light in peripheral FoV is therefore easier to face the mentioned large-angle non-uniformity issue. To acquire a decent FoV and light uniformity, usually two or three layers of waveguides with different grating pitches are adopted.

Regarding the system performance, the eyebox is generally large enough (~10 mm) to accommodate different user’s IPD and alignment shift during operation. A parameter of significant concern for a waveguide combiner is its FoV. From the *k*-vector analysis, we can conclude the theoretical upper limit is determined by the waveguide refractive index. But the light/color uniformity also influences the effective FoV, over which the degradation of image quality becomes unacceptable. Current diffractive waveguide combiners generally achieve a FoV of about 50°. To further increase FoV, a straightforward method is to use a higher refractive index waveguide. Another is to tile FoV through direct stacking of multiple waveguides or using polarization-sensitive couplers^79,193^. As to the optical efficiency, a typical value for the diffractive waveguide combiner is around 50–200 nit/lm^6,189^. In addition, waveguide combiners adopting grating out-couplers generate an image with fixed depth at infinity. This leads to the VAC issue. To tackle VAC in waveguide architectures, the most practical way is to generate multiple depths and use the varifocal or multifocal driving scheme, similar to those mentioned in the VR systems. But to add more depths usually means to stack multiple layers of waveguides together^194^. Considering the additional waveguide layers for RGB colors, the final waveguide thickness would undoubtedly increase.

**Fig. 14:**
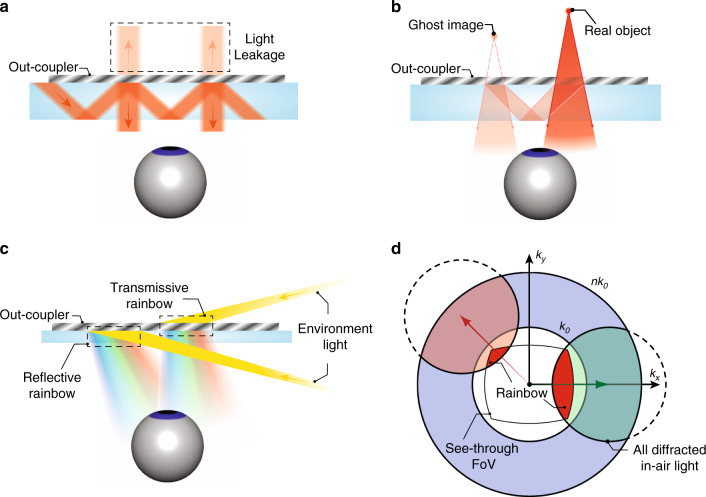
Artifacts of diffractive waveguide. Sketches of formations of **a** light leakage, **b** see-through ghost and **c** rainbow. **d** Analysis of rainbow formation with k-diagram

Other parameters special to waveguide includes light leakage, see-through ghost, and rainbow. Light leakage refers to out-coupled light that goes outwards to the environment, as depicted in Fig. 14a. Aside from decreased efficiency, the leakage also brings drawback of unnatural “bright-eye” appearance of the user and privacy issue. Optimization of the grating structure like geometry of SRG may reduce the leakage. See-through ghost is formed by consecutive in-coupling and out-couplings caused by the out-coupler grating, as sketched in Fig. 14b, After the process, a real object with finite depth may produce a ghost image with shift in both FoV and depth. Generally, an out-coupler with higher efficiency suffers more see-through ghost. Rainbow is caused by the diffraction of environment light into user’s eye, as sketched in Fig. 14c. The color dispersion in this case will occur because there is no cancellation of *k*-vector. Using the *k*-diagram, we can obtain a deeper insight into the formation of rainbow. Here, we take the EPE structure in Fig. 13a as an example. As depicted in Fig. 14d, after diffractions by the turning grating and the out-coupler grating, the *k* values are distributed in two circles that shift from the origin by the grating *k*-vectors. Some diffracted light can enter the see-through FoV and form rainbow. To reduce rainbow, a straightforward way is to use a higher index substrate. With a higher refractive index, the outer boundary of *k* diagram is expanded, which can accommodate larger grating *k*-vectors. The enlarged *k*-vectors would therefore “push” these two circles outwards, leading to a decreased overlapping region with the see-through FoV. Alternatively, an optimized grating structure would also help reduce the rainbow effect by suppressing the unwanted diffraction.

#### Achromatic waveguide

**Fig. 15:**
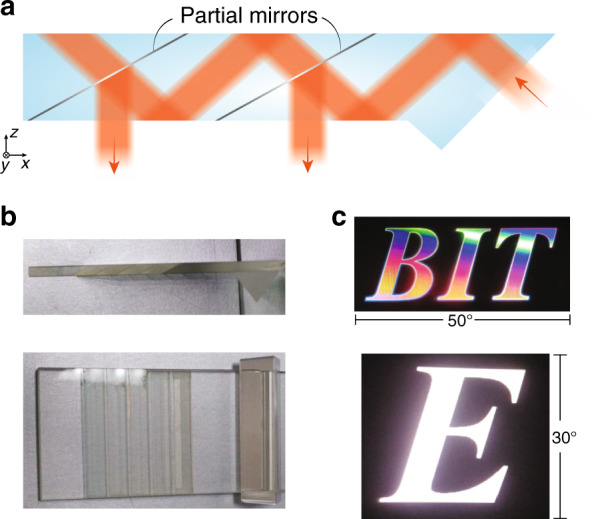
Geometric waveguide combiner. **a** Schematic of the system configuration. **b** Geometric waveguide with five partial mirrors. **c** Image photos demonstrating system FoV. Adapted from **b**, **c** ref. ^195^ with permission from OSA Publishing

Achromatic waveguide combiners use achromatic elements as couplers. It has the advantage of realizing full-color image with a single waveguide. A typical example of achromatic element is a mirror. The waveguide with partial mirrors as out-coupler is often referred as geometric waveguide^6,195^, as depicted in Fig. 15a. The in-coupler in this case is usually a prism to avoid unnecessary color dispersion if using diffractive elements otherwise. The mirrors couple out TIR light consecutively to produce a large eyebox, similarly in a diffractive waveguide. Thanks to the excellent optical property of mirrors, the geometric waveguide usually exhibits a superior image regarding MTF and color uniformity to its diffractive counterparts. Still, the spatially discontinuous configuration of mirrors also results in gaps in eyebox, which may be alleviated by using a dual-layer structure^196^. Wang et al. designed a geometric waveguide display with five partial mirrors (Fig. 15b). It exhibits a remarkable FoV of 50° by 30° (Fig. 15c) and an exit pupil of 4 mm with a 1D EPE. To achieve 2D EPE, similar architectures in Fig. 13a can be used by integrating a turning mirror array as the first 1D EPE module^197^. Unfortunately, the *k*-vector diagrams in Fig. 13b, d cannot be used here because the *k* values in *x-y* plane no longer conserve in the in-coupling and out-coupling processes. But some general conclusions remain valid, like a higher refractive index leading to a larger FoV and gradient out-coupling efficiency improving light uniformity.

**Fig. 16:**
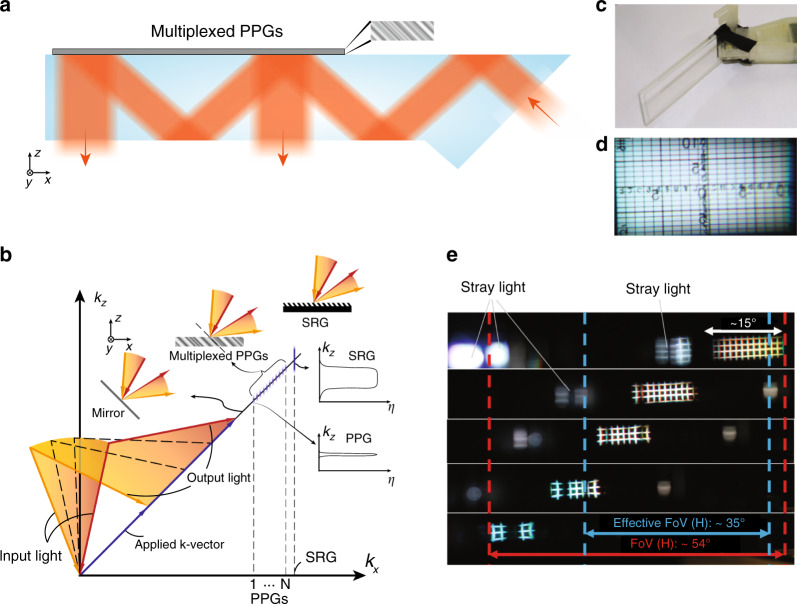
Skew-mirror waveguide combiner. **a** System configuration. **b** Diagram demonstrating how multiplexed PPGs resemble the behavior of a mirror. Photos showing **c** the system and **d** image. **e** Picture demonstrating effective system FoV. Adapted from **c**–**e** ref. ^199^ with permission from ITE

The fabrication process of geometric waveguide involves coating mirrors on cut-apart pieces and integrating them back together, which may result in a high cost, especially for the 2D EPE architecture. Another way to implement an achromatic coupler is to use multiplexed PPHOE^198,199^ to mimic the behavior of a tilted mirror (Fig. 16a). To understand the working principle, we can use the diagram in Fig. 16b. The law of reflection states the angle of reflection equals to the angle of incidence. If we translate this behavior to *k*-vector language, it means the mirror can apply any length of *k*-vector along its surface normal direction. The *k*-vector length of the reflected light is always equal to that of the incident light. This puts a condition that the *k*-vector triangle is isosceles. With a simple geometric deduction, it can be easily observed this leads to the law of reflection. The behavior of a general grating, however, is very different. For simplicity we only consider the main diffraction order. The grating can only apply a *k*-vector with fixed *k*_*x*_ due to the basic diffraction law. For the light with a different incident angle, it needs to apply different *k*_*z*_ to produce a diffracted light with equal *k*-vector length as the incident light. For a grating with a broad angular bandwidth like SRG, the range of *k*_*z*_ is wide, forming a lengthy vertical line in Fig. 16b. For a PPG with a narrow angular bandwidth, the line is short and resembles a dot. If multiple of these tiny dots are distributed along the oblique line corresponding to a mirror, then the final multiplexed PPGs can imitate the behavior of a tilted mirror. Such a PPHOE is sometimes referred as a skew-mirror^198^. In theory, to better imitate the mirror, a lot of multiplexed PPGs is preferred, while each PPG has a small index modulation *δn*. But this proposes a bigger challenge in device fabrication. Recently, Utsugi et al. demonstrated an impressive skew-mirror waveguide based on 54 multiplexed PPGs (Fig. 16c, d). The display exhibits an effective FoV of 35° by 36°. In the peripheral FoV, there still exists some non-uniformity (Fig. 16e) due to the out-coupling gap, which is an inherent feature of the flat-type out-couplers.

Finally, it is worth mentioning that metasurfaces are also promising to deliver achromatic gratings^200,201^ for waveguide couplers ascribed to their versatile wavefront shaping capability. The mechanism of the achromatic gratings is similar to that of the achromatic lenses as previously discussed. However, the current development of achromatic metagratings is still in its infancy. Much effort is needed to improve the optical efficiency for in-coupling, control the higher diffraction orders for eliminating ghost images, and enable a large size design for EPE.

Generally, achromatic waveguide combiners exhibit a comparable FoV and eyebox with diffractive combiners, but with a higher efficiency. For a partial-mirror combiner, its combiner efficiency is around 650 nit/lm^197^ (2D EPE). For a skew-mirror combiner, although the efficiency of multiplexed PPHOE is relatively low (~1.5%)^199^, the final combiner efficiency of the 1D EPE system is still high (>3000 nit/lm) due to multiple out-couplings.

Table 2 summarizes the performance of different AR combiners. When combing the luminous efficacy in Table 1 and the combiner efficiency in Table 2, we can have a comprehensive estimate of the total luminance efficiency (nit/W) for different types of systems. Generally, Maxwellian-type combiners with pupil steering have the highest luminance efficiency when partnered with laser-based light engines like laser-backlit LCoS/DMD or MEM-LBS. Geometric optical combiners have well-balanced image performances, but to further shrink the system size remains a challenge. Diffractive waveguides have a relatively low combiner efficiency, which can be remedied by an efficient light engine like MEMS-LBS. Further development of coupler and EPE scheme would also improve the system efficiency and FoV. Achromatic waveguides have a decent combiner efficiency. The single-layer design also enables a smaller form factor. With advances in fabrication process, it may become a strong contender to presently widely used diffractive waveguides.

**Table 2:** Comparison of different AR combiners

	Combiner efficiency	FoV (diagonal)	Eyebox	Form factor	Major challenge
Geometric optics	~4000 nit/lm	50°–120°^158^	~8 mm^158^	Large	Shrinking size, etendue conservation
Maxwellian-type	~50000 nit/lm (pupil st.) ~3000 nit/lm (pupil dup.)	~100°^143^	2–4 mm (single view) ~8 mm (pupil st./dup.)	Small	Implementation of pupil steering or duplication.
Diffractive waveguide	50–200 nit/lm^6^	~50°^6^	~15 mm	Medium (multilayer)	Combiner efficiency, FoV
Achromatic waveguide	650–1000 nit/lm (2D EPE)^197^ 1500–3000 nit/lm (1D EPE)^199^	~60°^199^	~15 mm	Small	Fabrication, FoV

## Conclusions and perspectives

VR and AR are endowed with a high expectation to revolutionize the way we interact with digital world. Accompanied with the expectation are the engineering challenges to squeeze a high-performance display system into a tightly packed module for daily wearing. Although the etendue conservation constitutes a great obstacle on the path, remarkable progresses with innovative optics and photonics continue to take place. Ultra-thin optical elements like PPHOEs and LCHOEs provide alternative solutions to traditional optics. Their unique features of multiplexing capability and polarization dependency further expand the possibility of novel wavefront modulations. At the same time, nanoscale-engineered metasurfaces/SRGs provide large design freedoms to achieve novel functions beyond conventional geometric optical devices. Newly emerged micro-LEDs open an opportunity for compact microdisplays with high peak brightness and good stability. Further advances on device engineering and manufacturing process are expected to boost the performance of metasurfaces/SRGs and micro-LEDs for AR and VR applications.

## Supplementary information

Supplementary Information

## Data Availability

All data needed to evaluate the conclusions in the paper are present in the paper. Additional data related to this paper may be requested from the authors.
