# Supplementary material for: Augmented reality and virtual reality displays: emerging technologies and future perspectives
Source: Light Sci Appl. 2021 Oct 25;10:216. doi: 10.1038/s41377-021-00658-8 (PMC8546092; doi:10.1038/s41377-021-00658-8)
Supplement: Supplementary file 1 — Supplementary Information [file 41377_2021_658_MOESM1_ESM.docx]

**Supplementary Information for Augmented reality and virtual reality displays: emerging technologies and future perspectives**

*Jianghao Xiong, En-Lin Hsiang, Ziqian He, Tao Zhan, and Shin-Tson Wu^*^*

*College of Optics and Photonics, University of Central Florida, Orlando, FL 32816, USA*

*Corresponding author: [swu@creol.ucf.edu](mailto:swu@creol.ucf.edu)

_____________________________________________________________________________

**Table S1 Parameters of gratings in the simulation**

|  | Refractive index | Grating pitch (Λ*_x_*) | Bragg pitch (Λ*_B_*) | Bragg surface slant angle | Grating thickness |
| --- | --- | --- | --- | --- | --- |
| Transmissive  PPHOE | *n* = 1.5, *δn* = 0.02 | 3000 nm | 3000 nm | 90º | 2500 nm |
| Reflective PPHOE |  |  | 190 nm | 7.3º | 1667 nm |
| Transmissive LCHOE | *n_o_* = 1.50  *n_e_* = 1.65 |  | 3000 nm | 90º | 12500 nm |
| Reflective LCHOE |  |  | 190 nm | 7.3º | 20000 nm |

**Calculation of luminous efficiency**

For blue micro-LEDs, we perform two calculations. First, from Table 1 in ref.^133^, we can obtain the brightness of 8,300 cd m^-2^, area of 0.384 cm^2^ and power consumption of 1.17 W. To calculate luminous flux, we assume the emission solid angle is 2*π*. Therefore, the total luminous flux is 8300×0.384×10^-4^×2*π* = 2.0 lm. The luminous efficacy is 1.71 lm W^-1^. Here, the choice of 2*π* solid angle mainly comes from the consideration of irregular angular emission profiles of micro-LEDs, especially for GB colors. The actual solid angle averaged by the emission profile is smaller than 2*π*. Therefore, the final calculated values only serve as a rough estimate.

Second, from Fig. 5 in ref.^131^, we can acquire the highest EQE of 6% for 20 µm-LED at current density of 5 A cm^-2^. To calculate WPE, we assume the wavelength of 450 nm and voltage of 2.75 V same as ref.^133^ (the voltage for 5 A cm^-2^ is hard to read from Fig. 2). The WPE is calculated as $WPE=EQE\frac{hc}{e\lambda V}=0.06\times\frac{1.24}{0.45\times2.75}=6\%$. With maximum luminous efficacy of radiation for 450 nm of around 33 lm W^-1^, we can obtain luminous efficacy of 1.98 lm W^-1^, which is close to the result calculated from ref.^133^.

For green micro-LEDs, we can read from Fig. 2 in ref.^132^ the brightness of a 25 µm LED to be 2×10^5^ cd m^-2^ under current density of 5 A cm^-2^. The luminous flus is 2×10^5^×(25×10^-6^)^2^×2*π* = 7.85×10^-4^ lm. From Fig. 1 we can read the voltage is around 2.3 V. The power consumption is calculated as 2.3×5×10^4^×(25×10^-6^)^2^ = 7.19×10^-5^ W. The luminous efficacy is 10.9 lm W^-1^.

For red micro-LEDs, from Fig. 15 in ref.^130^, we can read under current of 10 mA, the light output power is 330 µW, with a voltage of around 2.3 V. The WPE is calculated as 330×10^-6^/(10×10^-3^×2.3) = 1.4. With maximum luminous efficacy of radiation for 620 nm of 219 lm W^-1^. The luminous efficacy is calculated as 3.14 lm W^-1^.

Here, to calculate the luminous efficacy for white light, we assume the proportion of RGB lights is 3:6:1. Then the final luminous efficacy for RGB micro-LEDs is calculated as 10/(3/3.14 + 6/10.9 + 1/1.71) = 4.78 lm W^-1^. For QD micro-LED, if we assume the color conversion efficiency is 100%. Then the equivalent luminous efficacy for green (532 nm) and red (625 nm) light is 31.2 lm W^-1^ and 11.3 lm W^-1^. So the final luminous efficacy for white light is 10/(3/11.3 + 6/31.2 + 1/1.71) = 9.59 lm W^-1^.

From ref.^124,125^, the typical current efficiency is around 6 cd A^-1^. Due to the strong cavity of OLED, the angular emission is not Lambertian-type. Therefore, we loosen the solid angle in the range from *π* to 2*π*. If we use a voltage of 5 V, then the luminous efficacy is calculated as 3.77 lm W^-1^ for emission solid angle of *π* and 7.53 lm W^-1^ for 2*π*.

**Calculation of combiner efficiency**


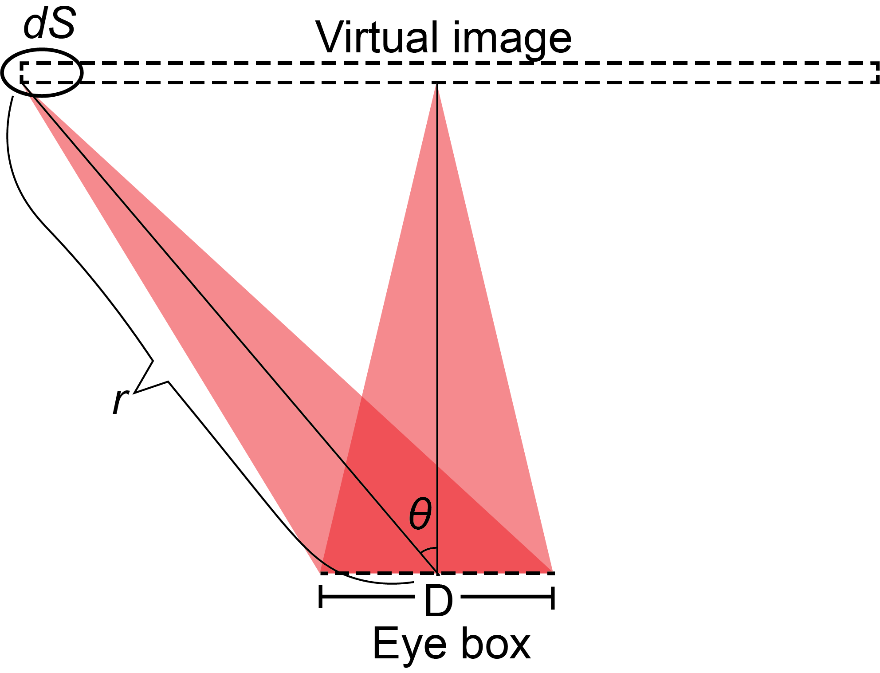


Fig. S1. Configuration of the combiner

To calculate the maximum combiner efficiency assuming no light waste, we can see the configuration in Fig. S1. The virtual image is assumed to be at depth *d*, the system eye box has a square shape with side length of *D*, the half FoV in one dimension is *θ*. We assume the luminance across the FoV is uniform (*L*). Then the total luminous flux *Φ* can be calculated as:

$\Phi=\int L\frac{D^{2}}{r^{2}}\cos\theta dS$ (S1)

The following relation can be derived:

$\Phi=D^{2}\Omega_{\text{FoV}}L$ (S2)

where $\Omega_{\text{FoV}}$is the solid angle of system FoV. Therefore, the luminance of image can be calculated by dividing input luminous flux by the product of eye box size and FoV solid angle. For a diagonal FoV of 60º (44º by 44º) and a 10 mm by 10 mm eye box, the luminance can be calculated as *L* = 1/(0.01^2^×4sin^-1^(sin^2^(22º))) = 17,756 nit. The maximum combiner efficiency is 17,756 nit lm^-1^. For a Maxwellian-type display, we take diagonal FoV of 100º (80º by 80º) and pupil diameter of 4 mm. Then the maximum combiner efficiency is 46,707 nit lm^-1^.
